# Characterization of *Vibrio cholerae’s* Extracellular Nuclease Xds

**DOI:** 10.3389/fmicb.2019.02057

**Published:** 2019-09-10

**Authors:** Katharina Pressler, Fabian Mitterer, Dina Vorkapic, Joachim Reidl, Monika Oberer, Stefan Schild

**Affiliations:** ^1^Institute of Molecular Biosciences, University of Graz, Graz, Austria; ^2^BioTechMed-Graz, Graz, Austria

**Keywords:** exonuclease, enzyme domains, enzyme properties, active-center, cholera

## Abstract

The Gram-negative bacterium *Vibrio cholerae* encodes two nucleases, Dns and Xds, which play a major role during the human pathogen’s lifecycle. Dns and Xds control three-dimensional biofilm formation and bacterial detachment from biofilms via degradation of extracellular DNA and thus contribute to the environmental, inter-epidemic persistence of the pathogen. During intestinal colonization the enzymes help evade the innate immune response, and therefore promote survival by mediating escape from neutrophil extracellular traps. Xds has the additional function of degrading extracellular DNA down to nucleotides, which are an important nutrient source for *V. cholerae*. Thus, Xds is a key enzyme for survival fitness during distinct stages of the *V. cholerae* lifecycle and could be a potential therapeutic target. This study provides detailed information about the enzymatic properties of Xds using purified protein in combination with a real time nuclease activity assay. The data define an optimal buffer composition for Xds activity as 50 mM Tris/HCl pH 7, 100 mM NaCl, 10 mM MgCl_2_, and 20 mM CaCl_2_. Moreover, maximal activity was observed using substrate DNA with low GC content and ambient temperatures of 20–25°C. *In silico* analysis and homology modeling predicted an exonuclease domain in the C-terminal part of the protein. Biochemical analyses with truncated variants and point mutants of Xds confirm that the C-terminal region is sufficient for nuclease activity. We also find that residues D787 and H837 within the predicted exonuclease domain are key to formation of the catalytic center.

## Introduction

*Vibrio cholerae*, a facultative human pathogen, is able to transit between two different environmental habitats, the aquatic reservoir and the human intestinal tract, causing the secretory diarrheal disease cholera (Colwell, 1996, 2004). Plants, zooplankton, crustaceans etc. present surfaces for formation of *V. cholerae* biofilms and allow its persistence in the aquatic reservoir (Colwell, 1996, 2004). During this phase of its lifecycle, bacterial aggregates detach from the biofilms and constitute an agent for initial infection of the human host via oral ingestion (Huq et al., 1990, 2008; Tamplin et al., 1990). To counter environmental challenges the bacterium adapts its physiology continuously through rapid changes in transcriptional regulation, protein synthesis and post-translational control. Proteolysis is particularly important during multiple stages of *V. cholera’s* lifecycle by varying virulence gene expression and biofilm growth, e.g., through control of transcription factors TcpP and ToxR; mucosal escape response or FliA degradation (Almagro-Moreno et al., 2015; Pressler et al., 2016; Wurm et al., 2017). Moreover, dramatic shifts in gene expression are essential for the survival of the bacteria within extreme environments like the acidic human stomach or the nutrient poor aquatic environment. Early induced genes initiate the virulence gene cascade leading to colonization of the small intestine and to severe diarrhea, while late induced genes increase fitness at the late infection state and are advantageous for the transition to the aquatic environment (Cash et al., 1974; Bennish, 1994; Schild et al., 2007). Two predicted extracellular nucleases of *V. cholerae*, encoded by *xds* (VC2621) and *dns* (VC0470), were shown to facilitate its survival fitness in- and outside of the host (Newland et al., 1985; Focareta and Manning, 1991; Schild et al., 2007).

Extracellular nucleases are known to control biofilm structure in several pathogens, e.g., *Staphylococcus*, *Neisseria*, *Shewanella*, through degradation of extracellular DNA (eDNA) (Godeke et al., 2011; Kiedrowski et al., 2011; Steichen et al., 2011). This matrix component is an abundant polymer in soil and water. Combined with *Vibrio* exopolysaccharides (VPS) and a variety of matrix proteins, it provides a major component of the *Vibrio* biofilm matrix. eDNA is reported to be important for the initial attachment of the bacteria to the surface. When combined with nucleases, it acts as a flexible structural component, which can be modified according to the needs of the bacteria. Deletion of both *V. cholerae* nucleases results in thick and disorganized biofilm that lacks the fluid-filled channels and pillars typically present for effective transport of molecules (Watnick and Kolter, 1999; Yildiz and Schoolnik, 1999; Watnick et al., 2001; Vlassov et al., 2007; Seper et al., 2011; Berk et al., 2012). Although both enzymes utilize DNA as substrate, their enzymatic reactions and regulation differ. While Dns is an EndA homolog that acts as an endonuclease, Xds exhibits exonuclease activity (Seper et al., 2011). Moreover, *dns* expression is repressed via the quorum sensing regulator HapR, while *xds* expression is controlled independently of population density. Notably, *dns* expression fluctuates during biofilm formation, whereas Xds increases over time to reach a maximum in mature biofilms (Seper et al., 2011). Dns seems to be the prominent nuclease for establishment of the three-dimensional (3D) biofilm structure, but Xds is essential for degradation of eDNA to the nucleotide level (Blokesch and Schoolnik, 2008; Seper et al., 2011). Expression of both nucleases in late stages of the mature biofilm indicates their importance during nutrient deprivation. Recently, it was shown that both nucleases are expressed under low phosphate conditions, which *V. cholerae* faces after the transition from the host to the aquatic environment. This is similar to reports of *Pseudomonas aeruginosa*, which represents another human pathogen that degrades eDNA down to carbon, nitrogen, and phosphate levels (Blokesch and Schoolnik, 2008; Pratt et al., 2009; Mulcahy et al., 2010; Seper et al., 2011). Additionally, *xds* expression is induced via the phosphate stress response regulator PhoB, as part of the two-component system PhoB/R, which is active upon phosphate limitation (McDonough et al., 2014). The activity of both nucleases results in degradation of eDNA and the extracellular accumulation of nucleotides. The latter are further transported via OmpK through the outer membrane followed by dephosphorylation via three periplasmic phosphatases. While free phosphate is taken up by the Pst/PhoU system the nucleosides are transported via the NupC system into the cell (Osborn and Wu, 1980; Seper et al., 2011; McDonough et al., 2015; Gumpenberger et al., 2016).

As biofilms serve as a reservoir for eDNA in the outside environment, neutrophil extracellular traps (NETs) provide a source of DNA inside the host. NETs originate from neutrophils upon contact with microbes as a first line of defense of the innate immune system (Fuchs et al., 2007). They consist of a nuclear or mitochondrial backbone associated with cytoplasmic and granula proteins. These components form fibers for efficient capture and killing of intruders effectively preventing the spread of microbes from the initial site of infection (Brinkmann et al., 2004; Papayannopoulos and Zychlinsky, 2009). We showed recently, that presence of eDNA, provided by NETs, induce both extracellular nucleases. Degradation of NETs by Xds and Dns, increases *V. cholerae* colonization fitness *in vivo* by liberation of the bacteria out of this entrapment (Seper et al., 2013).

Intriguingly, Xds homologs are rare yet often found in pathogens where biofilm formation is associated with virulence, e.g., *Acinetobacter baumannii* and *P. aeruginosa* (Allesen-Holm et al., 2006; Smith et al., 2007; Sahu et al., 2012; NCBI, 2015). Until today, no Xds homolog was found to be present in the human host highlighting the enzyme’s potential use for tailored therapy. In this context, highly specific Xds inhibitors would prevent dysbiosis of the human microbiome caused by standard antibiotic treatments. Herein we present an in depth characterization of Xds enzymatic properties in order to provide detailed understanding of its nuclease activity.

## Materials and Methods

### Bacterial Strains and Growth Conditions

Bacterial strains and plasmids used in this study are listed in Table 1; oligonucleotides are listed in Table 2. *V. cholerae* C6709, a spontaneous streptomycin (Sm)-resistant mutant of the clinical isolate O1 El Tor Inaba was used as wild type (WT) (Roberts et al., 1992), *Escherichia coli* strains DH5αλ*pir* and SM10λ*pir* were used for genetic manipulation (Kolter et al., 1978; Hanahan, 1983; Miller and Mekalanos, 1988), BL21 (NEB) for expression of proteins, *Cutibacterium acnes* (ATCC 6919; IA1) and *Haemophilus influenzae* (RD KW20) as template for dsDNA with 67 and 31% GC content, respectively (see Table 1). If not noted otherwise, strains were cultured in Luria Bertani (LB) broth (1% tryptone, 1% NaCl, 0.5% yeast extract) either shaking with 180 rpm or on LB broth agar plates with aeration at 37°C. If required, antibiotics or other supplements were used in the following final concentrations: streptomycin (Sm) 100 μg/ml, ampicillin (Ap) 100 μg/ml or 50 μg/ml in combination with other antibiotics, isopropyl-β-thiogalactopyranoside (IPTG) 0.5 mM, and glucose (Gluc) 0.2%.

**TABLE 1:** Strains and plasmids used in this study.

**Strain or plasmid**	**Genotype, resistance, description**	**References**
*E. coli*		
DH5aλpir	F^–^Φ80Δ*lacZ*Δ*M15*Δ(*argFlac*)*U169 deoR recA1 endA1 hsdR17* (r_K_^–^m_K_^+^) *supE44 thi-1 gyrA69 relA1*, λ*pir*R6K, Ap^*r*^	Hanahan (1983)
SM10λpir	thi thr leu tonA lacY supE recA:RPA-2-Te:Mu λpirR6K, Km^r^	Miller and Mekalanos (1988)
BL21 (DE3)	*fhuA2 [lon] ompTgal* (λ *DE3) [dcm]*Δ*hsdS* λ *DE3* = λ*sBamHIo*Δ*Eco*RI*-B int:(lacI:PlacUV5:T7 gene1) i21*Δ*nin5*	NEB
*V. cholerae*		
WT	C6709, wild type *V. cholerae* strain serogroup: O1; biotype: El Tor; serotype: Inaba; Peru 1991,*tcpA* + *ctx* + *hapR* + spontaneous Sm^R^	Roberts et al. (1992)
C6709Δ*xds*Δ*dns*	deletion of VC2621 and VC0470 in C6709,Sm^R^	Seper et al. (2011)
Δ*dns*	Deletion of *dns*(VC0470)in C6709, Sm^R^	Seper et al. (2011)
Δ*epsC-N*	Deletion of *epsC-N* (VC2734-VC2723) in C6709, Sm^R^	This study
Δ*dns*Δ*epsC-N*	Deletion of *dns*(VC0470) *and epsC-N* (VC2734-VC2723) in C6709, Sm^R^	This study
*C. acnes*	ATCC 6919; IA1; Isolated from: acne patient; Source: DSMZ	Douglas and Gunter (1946)
*H. influenzae*	Rd KW20; Un encapsulated variant of a former capsular serotype d strain, obtained from A. Wright	Wilcox and Smith (1975)
Plasmids		
pRS415	Yeast and bacterial Plasmid; Ap^R^	Stratagene
pCVD442	*ori6K mobRP4 sacB*, Ap^*r*^	Donnenberg and Kaper (1991)
pAC1000	Cm^R^	Hava et al. (2003)
p	pTRC99a, IPTG-inducible vector; Ap^R^	Tamayo et al. (2010)
pxds	Xds from C6709 with C terminal FLAG tag in pTRC99a	This study
pxds^Δ^*^*S*39^*^–^*^*G*137^*	Deletion of S39-G137 of Xds, with C terminal FLAG tag on the pTRC99A, Ap^R^	This study
pxds^Δ^*^*S*39^*^–^*^*Q*159^*	Deletion of S39-Q159 of Xds, with C terminal FLAG tag on the pTRC99A, Ap^R^	This study
pxds^Δ^*^*S*39^*^–^*^*S*184^*	Deletion of -S39-S184 of Xds, with C terminal FLAG tag on the pTRC99A, Ap^R^	This study
pxds^Δ^*^*S*39^*^–^*^*I*200^*	Deletion of S39-I200of Xds, with C terminal FLAG tag on the pTRC99A, Ap^R^	This study
pxds^Δ^*^*S*39^*^–^*^*V*233^*	Deletion of S39-V233 of Xds, with C terminal FLAG tag on the pTRC99A, Ap^R^	This study
pxds^Δ^*^*S*39^*^–^*^D248^*	Deletion of S39-D248 of Xds, with C terminal FLAG tag on the pTRC99A, Ap^R^	This study
pxds^Δ^*^*S*39^*^–^*^*G*473^*	Deletion of S39-G473 of Xds, with C terminal FLAG tag on the pTRC99A, Ap^R^	This study
pxds^Δ^*^*L*30^*^–^*^*G*134^*	Deletion of L30-G134 of Xds, with C terminal FLAG tag on the pTRC99A, Ap^R^	This study
pxds^Δ^*^*L*30^*^–^*^*S*184^*	Deletion of L30-S184 of Xds, with C terminal FLAG tag on the pTRC99A, Ap^R^	This study
pxds^Δ^*^*L*30^*^–^*^*I*200^*	Deletion of L30-I200of Xds, with C terminal FLAG tag on the pTRC99A, Ap^R^	This study
pxds^Δ^*^*N*221–Q300^*	Deletion of N221-Q300 of Xds, with C terminal FLAG tag on the pTRC99A, Ap^R^	This study
pxds^Δ^*^*L*30^*^–^*^*G*473^*	Deletion of L30-G473 of Xds, with C terminal FLAG tag on the pTRC99A, Ap^R^	This study
pxds^D787A^	Point mutation in D787 to Aof Xds, with C terminal FLAG tag on the pTRC99A, Ap^R^	This study
pxds^H837A^	Point mutation in H837 to Aof Xds, with C terminal FLAG tag on the pTRC99A, Ap^R^	This study
pxds^C188A^	Point mutation in C188 to A of Xds, with C terminal FLAG tag on the pTRC99A, Ap^R^	This study
pxds^C276A^	Point mutation in C276 to Aof Xds, with C terminal FLAG tag on the pTRC99A, Ap^R^	This study
pxds^C661A^	Point mutation in C661 to A of Xds, C terminal FLAG tag on the pTRC99A, Ap^R^	This study
pxds^C684A^	Point mutation in C684 to A of Xds, C terminal FLAG tag on the pTRC99A, Ap^R^	This study

**TABLE 2:** Oligonucleotides used in this study.

**Primer name**	**Sequence (5′ to 3′)**
Xds_*Eco*RI_1	AAAGAATTC GAAATGAGAACAACCCC
Xds_XbaI_2	AAATCTAGA CTATTTGTCATCGTCGTCCTTGTAGTCGCG ACGGCGACGCCAAA
S39-G137_3	TCTTTCGCAAAATCTGCGCCGCCTTCAACGTATTGAG AAATC
S39-G137_4	GATTTCTCAATACGTTGAAGGCGGCGCAGATTTTGCG AAAGA
S39-Q159_3	GCCTTGGGTCGCCCAATCGGAAGCGCCTTCAACGTATT GAGAAAT
S39-Q159_4	ATTTCTCAATACGTTGAAGGCGCTTCCGATTGGGCGA CCCAAGGC
S39-S184_3	ACCATCTAGCGTACAATTGAAGGGCCTTCAACGTATTGA GAAAT
S39-S184_4	ATTTCTCAATACGTTGAAGGCTTCAATTGTACGCTAG ATGGT
S39-I200_3	GCCTTCACCTTGAATTTGTTGGCCTTCAACGTATTGA GAAAT
S39-I200_4	ATTTCTCAATACGTTGAAGGCCAACAAATTCAAGGTG AAGGC
S39-V233_3	GCCTTTAGTCAGTCCTGTCGTGCCTTCAACGTATTGA GAAAT
S39-V233_4	ATTTCTCAATACGTTGAAGGCACGACAGGACTGACTA AAGGC
S39-D248_3	TTCAGAGGTATTCGGGTTGTAGTCGCCTTCAACGTATT GAGAAAT
S39-D248_4	ATTTCTCAATACGTTGAAGGCGACTACAACCCGAATA CCTCT
S39-G473_3	GTTGAACGTGGCAATGCGCAGATCGCCTTCAACGTATT GAGAAAT
S39-G473_4	ATTTCTCAATACGTTGAAGGCGATCTGCGCATTGCCACGT TCAAC
L30-G134_3	AAAATCTGCGCCGCCCATACTGTCAGCGTAGCTTGGC GCCAC
L30-G134_4	GTGGCGCCAAGCTACGCTGACAGTATGGGCGGCGCAG ATTTT
L30-S184_3	ACCATCTAGCGTACAATTGAAGGCGTCAGCGTAGCTTG GCGCCAC
L30-S184_4	GTGGCGCCAAGCTACGCTGACGCCTTCAATTGTACGCT AGATGGT
L30-I200_3	GCCTTCACCTTGAATTTGTTGGTCAGCGTAGCTTGGC GCCAC
L30-I200_4	GTGGCGCCAAGCTACGCTGACCAACAAATTCAAGGTG AAGGC
N221-Q300_3	GCGCAGCTTGCTGGCC GGTGATGTAGGGATA
N221-Q300_4	TATCCCTACATCACC GGCCAGCAAGCTGCG
L30-G473_3	GTTGAACGTGGCAATGCGCAGATCGTCAGCGTAGCTTG GCGCCAC
L30-G473_4	GTGGCGCCAAGCTACGCTGACGATCTGCGCATTGCCAC GTTCAAC
D787A_3	CTGACTAACAGATG**AGC**CAACGCGCCGACTT
D787A_4	AAGTCGGCGCGTTG**GCT**CATCTGTTAGTCAG
H837A_3	AGTACCGCTGGGTC**AGC**ATCTGAGGCGCGGAA
H837A_4	TTCCGCGCCTCAGAT**GCT**GACCCAGCGGTACT
C188A_3	TTCAGCACCATCTAGCGT**AGC**ATTGAAGGCACTCGG
C188A_4	CCGAGTGCCTTCAAT**GCT**ACGCTAGATGGTGCTGAA
C276A_3	CTGCACTTTGCCTTTCACG**GCC**ACCACATCGCCCGG
C276A_4	CCGGGCGATGTGGT**GGC**CGTGAAAGGCAAAGTGCAG
C661A_3	CGCGGCATCTTCCCA**AGC**CGCTGATCCTTTCGATTT
C661A_4	AAATCGAAAGGATCAGCG**GCT**TGGGAAGATGCCGCG
C684A_3	GACGCGGAAGTTTTC**AGC**TGCGCCTTGGTAATCGAG
C684A_4	CTCGATTACCAAGGCGCA**GCT**GAAAACTTCCGCGTC
CAT_BamHI_Fw	TTTGGATCC GATAAGCTTGATGAAAATTTGT
CAT_BamHI_Rev	TTAGGATCC GGTTAGTGACATTAGAAAA
HI_Fw	TTTTAAATGTTCCTTATTTATTAA
HI_ Rev	TATTGCTTACAATAGGAAATACAGA
CA_ Fw	GTGTTGACGCGCATGTCACAGCT
CA_ Rev	GTAGACGCCGGGAGCGACCCGGC
VC2734_SacI_1	AAAGAGCTC GCCTTGCTTAGGTTCA
VC2734_BamHI_2	TTAGGATCC CATAAATTTCCACGTTATTCC
VC2723_*Eco*RI_3	AATGAATTC TAGGATGTGTAATCCCATTCA
VC2373_XbaI_4	TTTTCTAGA TCATTCGCTGGCCTTTA
Seq_pTrc_Fw	TTGTGAGCGGATAACAA
Seq_pTrc-Rev	TCAGGCTGAAAATCTTCTCTC
Seq_1180nt_Fw	GAATCGGATGCCAAAGCACCAGAT

*^*a*^Restriction sites are underlined, point-mutations are in bold.*

### Construction of in Frame Mutants and Expression Plasmids

PCR conditions, the isolation of chromosomal DNA, plasmids or PCR products, and construction of expression plasmids were carried out as described previously (Seper et al., 2011). Qiaquick^®^ Gel extraction and Qiaquick^®^ PCR Purification kits (Qiagen) were used for purifying PCR products and digested plasmid DNA. PCR reactions for sub-cloning were carried out using the Q5^®^ High-Fidelity DNA Polymerase (NEB), while Taq DNA Polymerase (NEB) was used for all other PCRs. Deletion mutants were generated using derivatives of the suicide vector pCVD442 in combination with an established method (Donnenberg and Kaper, 1991). Respective suicide vectors were constructed by PCR-amplification of approximately 800 bp fragments, representing upstream and downstream regions of the gene of interest, using the oligonucleotide pairs X_Y_1 and X_Y_2 or X_Y_3 and X_Y_4, where X represents the gene and Y the restriction site/enzyme used (Table 2). Subsequently, fragments generated were digested with the appropriate restriction enzyme indicated by the name of the oligonucleotide, and finally ligated into an identically digested suicide plasmid pCVD442. The respective suicide plasmids were first transformed into *E. coli* DH5αλ*pir*. Positive clones were selected via PCR (data not shown) and further transformed in *E. coli* Sm10λ*pir* and then transferred into *V. cholerae* via conjugation. Cells were grown on Sm- and Ap-containing agar plates to select for the integration of the plasmid into the chromosome. This selection was followed by growth on sucrose to obtain Ap^s^ colonies, in which an excision of the plasmid from the chromosome took place. Correct deletions were confirmed by PCR (data not shown). Point mutants harboring an amino acid (AA) exchange as well as truncated versions of Xds were generated by SOE (splicing by overlap extension) PCR, using chromosomal DNA of *V. cholerae* WT as template and the oligonucleotide pairs Xds_*Eco*RI_1 and Xds_XbaI_2 as well as the oligos XY_3 and XY_4, where XY stands for the respective AA (Table 2) (Horton et al., 1989). The generation of overlapping regions allowed the annealing of the two PCR fragments in a further PCR reaction. The respective PCR fragments were digested with the appropriate restriction enzymes and ligated into a similarly digested, IPTG-inducible plasmid (pTRC99a). Ligation products were transformed into DH5αλpir and Ap^R^ colonies were characterized by PCR for the positive constructs which were verified by Sanger-sequencing (data not shown). Plasmids were isolated and transformed in to *V. cholerae* or *E. coli* strains, which were then tested for nuclease activity with DNase Agar plates, gel electrophoresis or nuclease activity assay with SYBR Green I (SGI). Further the clones were taken for cell fractionation or immunoblot analysis.

### Generation of Whole Cell Lysates (WCL)

To obtain WCL appropriate amounts of *V. cholerae* and *E. coli* cultures grown overnight (ON) were inoculated in fresh LB to an OD_600_ of 0.1, grown to an OD_600_ of 0.5 following induction with IPTG (0.5 mM) for 4 h with aeration at 37°C, 180 rpm shaking. Cell equivalents reflecting 1 ml of an OD_600_ of 1.3, were harvested by centrifugation in an Eppendorf centrifuge (5 min at 5,000 *g*), resuspended in 100 μl of Laemmli buffer, boiled for 30 min at 100°C and either stored at −20°C or directly used for SDS-PAGE. For immunoblot analyses of WCL the overall protein contents were assessed to contain similar protein levels by SDS-PAGE following Kang staining.

### Cellular Fractionation

Cellular fractionation was performed as described by Neu and Heppel (Neu and Heppel, 1965). *V. cholerae* strains grown ON were inoculated in fresh LB to an OD_600_ of 0.1, followed by induction with 0.5 mM IPTG at an OD_600_ of 0.5 for 4 h. Cell equivalents reflecting 1 l of a culture with OD_600_ of 1 were pelleted by centrifugation (10 min, 5,000 *g*) and washed in buffer (20 mM Tris/HCl pH 8). After a second centrifugation step, cells were resuspended in 20% sucrose and 1 mM Na-EDTA and incubated for 10 min subsequently shaking. After centrifugation for 10 min, 5,000 *g* at 4°C cells were resuspended in ice cold 0.5 mM Mg_2_SO_4_. Combination of cold temperature and hypotonic solution caused the burst of the bacterial outer membrane and release of the periplasmic fraction (PF) in the supernatant. Proteins in the PF were precipitated using trichloroacetic acid (TCA)/acetone for immunoblot analyses. The remaining pellet was further resuspended in 10 mM Tris/HCl pH 8 and lysed using 0.1 mm glass beads in combination with a PowerLyzer^TM^ 24 (MO BIO Laboratories, Inc.), applying three 1 min cycles at 3,400 rpm with 1 min intervals on ice between each cycle (Roier et al., 2012, 2013). Lysates were centrifuged for 30 min at 10,000 *g* and 4°C to obtain the cytoplasmic fraction (CF) represented by the supernatant. The remaining pellet was resuspended in 100 μl 10 mM Tris/HCl pH 8 and used as membrane fraction (MF).

### Precipitation of Proteins Using TCA/Acetone

Protein precipitation using TCA and acetone was essentially performed as described by Link and LaBaer (Link and Labaer, 2011), with following modifications. Solution of 100% TCA was added to the protein samples (PF) to the final TCA concentration of 20%. Samples were mixed and stored ON at −20°C. On the next day, samples were thawed and centrifuged 30,000 *g* for 1 h. Supernatant was carefully decanted and 20 ml of acetone was added to the pellet. Samples were again centrifuged 30,000 *g* for 1 h. A second washing step with 20 ml of acetone was performed with the final centrifugation step of 50,000 *g*. After decanting the supernatant, pellets were air-dried for 30 min and subsequently resuspended in 200 μl of TBS buffer. In attempt to completely dissolve the pellet, samples were placed in a sonification bath for 30 min. Undissolved particles were removed by short centrifugation.

### SDS-PAGE and Immunoblot Analysis

Proteins concentrations were generally determined with Bradford assay (BioRad) to ensure loading of equal protein amounts. To separate proteins the standard sodium dodecyl-sulfate-polyacrylamide gel electrophoresis (SDS-PAGE) procedure in combination with 12% gels and the PageRuler^TM^ Prestained Protein Ladder (Thermo Scientific) as a molecular mass standard was used. Proteins were stained according to Kang et al. (2002) or transferred to a nitrocellulose membrane (Amersham) for immunoblot analysis. Blocking was done overnight at 4°C in TBS (0.5 M Tris/HCl pH 7.5, 1.5 M NaCl) supplemented with 10% milk powder. FLAG-tagged proteins were detected with HRP-conjugated α-FLAG antibody (A8592, Sigma). After washing (5 × 5 min) with 1× TBS T (0.5 M Tris/HCl pH 7.5, 1.5 M NaCl, 0.5% Tween-20) blots were detected with ECL (Bio-Rad) according to the manufacturer’s instructions.

### Purification of Proteins by Affinity Chromatography

Agarose resin conjugated to anti-FLAG antibody M2 (A2220, Sigma) was used for protein purification. Proteins were extracted by main cultures inoculated and expressed as described above. Cells were harvested by centrifugation for 10 min at 4°C and 9,500 *g*, the cell pellet was re-suspended in lysis buffer (50 mM Tris/HCl pH 7.4, 100 mM NaCl and 1% TritonX) and lysed by sonication on ice. Cell lysates were clarified by centrifugation for 30 min at 4°C and 17,000 *g* and filtered with a syringe filter (0.45 μm). The proteins were purified using an agarose resin conjugated to anti-FLAG antibody M2 (A2220, Sigma) for 1.5 h, 4°C rotating. The resin was washed four times with 1 mL TBS for 5 min, 1,000 *g*, 4°C. Elution was carried out by 200 μl TBS containing 150 μg/ml of 3 × FLAG peptide (F4799, Sigma) and subsequently shaking at 4°C for 1 h. Further the resin was centrifuged at 8,200 *g* for 3 min and the supernatant was taken as protein solution. The concentrations of purified proteins were obtained by the absorbance at 280 nm using the NanoDrop^®^ ND-1000 Spectrophotometer (PEQLAB Biotechnologie GmbH, Erlangen, Germany).

### Homology Modeling

Homology model of the nuclease domain of Xds protein structure was modeled with the Phyre2 server using the intensive search mode (Kelley et al., 2015). Three templates (4ruw: 20% s.id, 4zkf: 13% s.id., 3ngo: 13% s.id.) were selected to model 94% of the residues at >90% confidence.

### DNase Agar Activity Test

DNase test agar (BD) was used according to manufactures protocol. Plates containing 0.5 mM IPTG and 100 μg/ml Ap were inoculated with respective strains and incubated at 37°C for 48 h before the standard HCl-DNA precipitation method for detection of DNA was used (Jeffries et al., 1957).

### DNase Activity Assay Using Agarose Gel Electrophoresis

DNase activity assay using a defined PCR fragment was performed as described previously (Blokesch and Schoolnik, 2008; Mulcahy et al., 2010). Bacterial strains were grown and induced with 0.5 mM IPTG for 4 h as described above. Cells were pelleted by centrifugation at 5,000 *g* for 5 min and supernatants were used for incubation at RT for 8 and 16 h, respectively. A 600 ng PCR fragment [1 kb fragment of the *cat* gene amplified from pAC1000 (Table 1) using oligonucleotides CAT_BamHI_Fw and CAT_BamHI_Rev (Table 2)] was used as a substrate in combination with 5× buffer (50 mM Tris/HCl pH7, 100 mM NaCl, 10 mM MgCl_2_, and 20 mM CaCl_2_). Finally, samples were visualized on agarose gels (0.8%).

### Real Time DNase Activity Assay Using SYBR Green I (SGI)

Nuclease assay with SGI nucleic acid stain (Invitrogen) was performed similar than described by Deng et al. (2012) and Zheng et al. (2013)As the truncated Xds-version. Briefly a 100 bp DNA fragment amplified by Xds_*Eco*RI_1 and His837Ala_3 was used as linearized dsDNA (standard 53% GC content) (see Table 2), whereas pRS415 served as template for circular dsDNA. For determination of Xds activity on DNA with variation in GC content, the 100 bp fragment was amplified using *C. acnes* chromosomal DNA with CA_Fw and CA_Rev (67% GC), and *H. influenzae* chromosomal DNA with HI_Fw and HI_Rev (31% GC), respectively (see Tables 1, 2). The substrate DNA was stained with SGI (1.000×) for 1 h at RT. FLAG purified protein (0.5 μg) was incubated with 220 ng of stained DNA substrate and 10 μl of buffer (different compositions). The reaction volume was filled up with distilled water to a total of 50 μl. Fluorescence measurements were carried out with a CFX96 Real-Time PCR Detection System (Bio-Rad).

Parameters deduced from the nuclease assay are area under curve (AUC), substrate degradation, duration to degrade 50% of the substrate and endpoint measurement of the remaining substrate in percent. AUC was calculated using GraphPad Prism software (La Jolla, CA, United States) by selecting “Analysis” and then “Area under curve” using 0.0 as a baseline for the *y*-axis. Substrate degradation was calculated in the linear range predicted between 60 and 80% fluorescence activity.

### Statistical Analysis

Data were analyzed using the Mann-Whitney U test or a Kruskal-Wallis test followed by *post hoc* Dunn’s multiple comparisons. Differences were considered significant at *P* values of <0.05. GraphPad Prism version 6 was used for all statistical analyses.

## Results

### Bioinformatical Analysis of Xds

**FIGURE 1:**
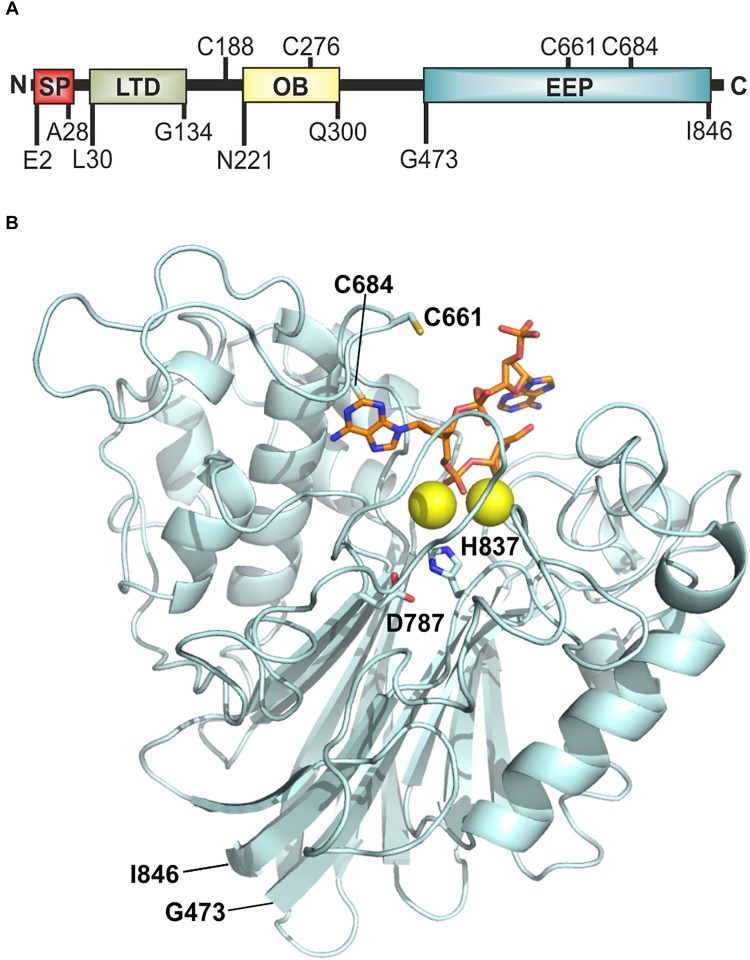
Model of Xds. **(A)** Graphical illustration of conserved domains of Xds. Residue numbers of domain boundaries are indicated below and cysteine residues are indicated above the peptide. **(B)** Homology model of the extracellular nuclease Xds from G473-I846 (pale cyan) using the templates 4ruw, 3ngo, 4zkf (Wang et al., 2014; Wu et al., 2014; Kim et al., 2017). Interaction of Xds with nucleic acid (orange) is shown using CNOT6L as reference (3ngo) (Wang et al., 2010). Active site is likely formed by D787 and H837 (indicated as sticks) according to catalytic residues of the templates 4zkf and 3ngo. Mg^2+^ ions in the active site are depicted as yellow spheres. Cysteine residues C661 and C684 are shown as yellow sticks. The model was prepared using Phyre2 (Kelley et al., 2015) and depicted using the PyMOL Molecular Graphics System, Version 2.2.0 Schrödiger, LLC.

Open reading frame VC2621 (UniProt: Q9KNV9) of *V. cholerae* O1 El Tor comprises 2610 bp, which encode the 869 amino acid (AA) protein Xds. The predicted molecular weight is 94.3 kDa (KEGG, 2017). Analysis with SignalP-5.0 software revealed a signal peptide with a cleavage probability of 0.9866 between Ala28 and Asp29, consistent with its extracellular activity (Seper et al., 2011). AA sequence analysis and 3D homology modeling indicated a Lamin-tail domain (LTD) (L30-G134) in the N-terminal part of the protein (Figure 1A) (Interpro, 2019; Mitchell et al., 2019). LTDs are usually found in the C-terminal part of nuclear lamins, which are intermediate filament proteins important for maintenance of cellular integrity. LTDs harbor an immunoglobulin fold and are suggested to be in involved in tethering proteins to membranes in bacteria (Fuchs and Yang, 1999; Krimm et al., 2002; Mans et al., 2004). Additionally, the sequence revealed an OB (oligonucleotide/oligosaccharide binding)-fold from N221-Q300 (Figure 1A). OB-folds can be found in both eukaryotic and prokaryotic proteins and are shown to form protein-DNA, -RNA or -protein interactions (Murzin, 1993; Arcus, 2002; Theobald et al., 2003). Finally, an endonuclease/exonuclease/phosphatase (EEP) domain is located at position G473-I846, which is predicted to hydrolyze the phosphodiester bonds of the nucleotides (Figure 1). A 3D model of the putative nuclease domain (G473-I846) was calculated using Phyre2 based on unpublished structures with Protein Data Bank (PDB)-IDs 4zkf and 4ruw and a nuclease domain (PDB-ID 3ngo) as templates (Figure 1B) (Wang et al., 2010; Kelley et al., 2015). The alignment identified D787 and H837 as potential catalytic residues when compared with other nuclease sequences. Comparisons of the templates showed residues E240, D410, N412, and H529 of 3ngo and residues E351, D496, N498, and H599 of 4zkf to be at equivalent positions as the Xds residues E532, D711, N713, and H837, respectively. These represent metal binding residues in the cleft of the active site. In addition, Xds harbors four cysteines C188, C276, C661, and C684. *In silico* analyses including 3D modeling did not predict a disulfide-bond within the nuclease domain (Ceroni et al., 2006). Other potential disulfide bonds could not be predicted, as the lack of a suitable template prevents homology modeling of the entire protein.

### Elucidation of Optimum Buffer Conditions for Xds

To investigate the activity of the extracellular nuclease, Xds was purified as C-terminal FLAG-tagged protein using the pTRC99a expression system and *E. coli* BL21 as a host. The nuclease assay was performed using 5.1 pmol of purified full-length Xds and 100 kb of double stranded DNA (dsDNA) fragment stained with SGI as a substrate. Degradation of DNA through hydrolysis of phosphodiester bonds to nucleotides was detected by a decrease in fluorescence excited from SGI bound to dsDNA. This fluorescent-based real time nuclease assay was used to identify the optimal reaction conditions, including concentrations of NaCl, MgCl_2_, CaCl_2_, as well as pH and temperature.

**FIGURE 2:**
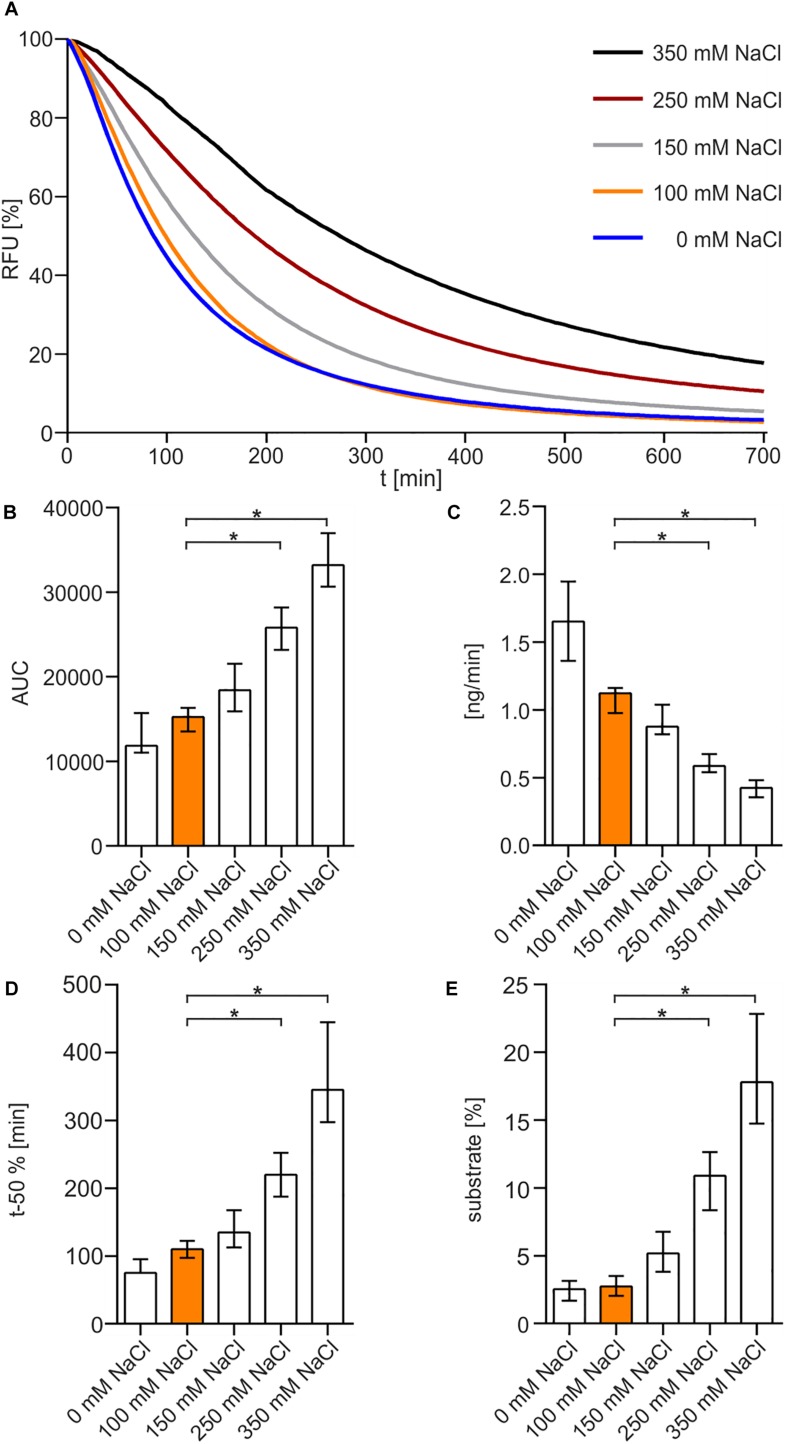
NaCl-dependency of Xds activity. **(A)** Shown are the relative fluorescence units (RFU) in percent indicating SGI bound to dsDNA. 5.1 pmol of purified Xds was incubated with 220 ng dsDNA (substrate) in Tris/HCl buffer (50 mM, pH7) at 25°C with different salt concentrations, 350 mM NaCl (black), 250 mM NaCl (red), 150 mM NaCl (gray), 100 mM NaCl (orange), and 0 mM NaCl (blue). Fluorescence was measured every 5 min for 12 h. **(B–E)** Bar charts summarize the enzyme parameters retrieved from the nuclease assays provided in panel A. Shown are the area under the curve (AUC, **B**), nanogram of substrate degraded per minute **(C)**, duration to degrade 50% of the substrate **(D)**, endpoint measurement of the remaining substrate in percent **(E)**. The data is presented as median from at least nine independent experiments. Error bars indicate the interquartile range. Significant differences to 100 mM NaCl are indicated by an asterisk (*P* < 0.05 Kruskal-Wallis test followed by *post hoc* Dunn’s multiple comparison).

To begin with, fastest degradation of DNA was shown for 0 and 100 mM NaCl, whereas higher amounts of NaCl resulted in a decrease in enzyme activity (Figure 2A). The following analysis measuring the area under the curve (AUC) showed a significant difference between 100 mM NaCl when compared to 250 and 350 mM NaCl (Figure 2B). Substrate degradation was evaluated within the linear range predicted between 60 and 80% fluorescence activity. The analysis revealed that Xds degrades 1.7 ng of DNA per minute at 0 mM NaCl and about 1.1 ng DNA per minute at 100 mM NaCl (Figure 2C). Further the enzyme takes 75 min at 0 mM NaCl and 110 min at 100 mM NaCl to degrade 50% of the substrate, respectively (Figure 2D). Endpoint measurement of the remaining substrate (Figure 2E) confirmed that the enzyme was most active at concentration of 0 and 100 mM NaCl.

**FIGURE 3:**
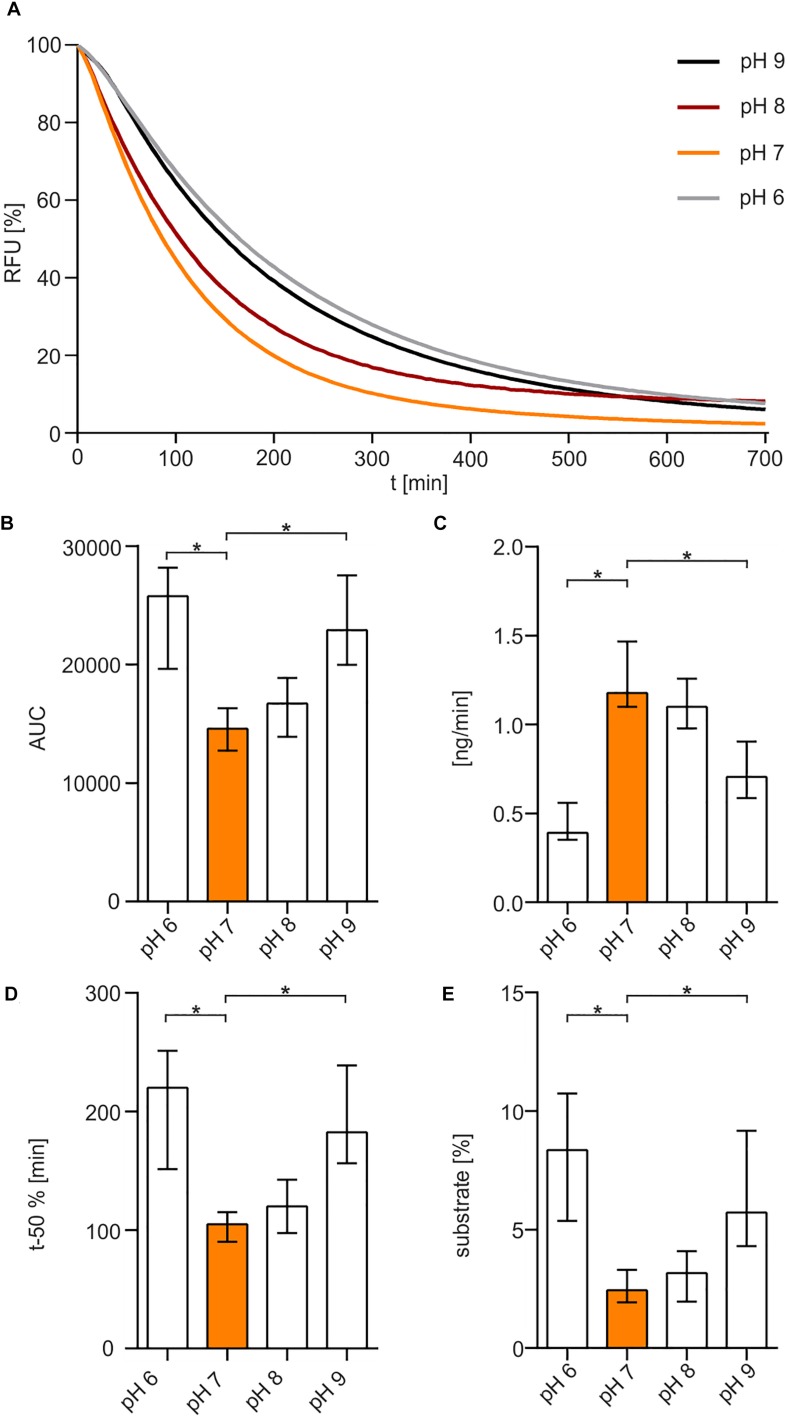
pH-dependency of Xds activity. **(A)** Shown are the relative fluorescence units (RFU) in percent indicating SGI bound to dsDNA. 5.1 pmol of purified Xds was incubated with 220 ng dsDNA (substrate) in Tris/HCl buffer (50 mM) and 100 mM NaCl at 25°C with variation in pH, pH 9 (black), pH 8 (red), pH 7 (orange), and pH 6 (gray). Fluorescence was measured every 5 min for 12 h. **(B–E)** Bar charts summarize the enzyme parameters retrieved from the nuclease assays provided in panel A. Shown are the area under the curve (AUC, **B**), nanogram of substrate degraded per minute **(C)**, duration to degrade 50% of the substrate **(D)**, endpoint measurement of the remaining substrate in percent **(E)**. The data is presented as median from at least 18 independent experiments. Error bars indicate the interquartile range. Significant differences to pH 7 are indicated by an asterisk (*P* < 0.05 Kruskal-Wallis test followed by *post hoc* Dunn’s multiple comparison).

The optimum pH for Xds was determined in buffer solutions containing 50 mM Tris/HCl (Figure 3A). The enzyme showed maximum activity at pH 7 to 8. Increasing the pH to 9 or decreasing it to 6 reduced Xds activity. This was also confirmed by measuring the enzyme activity in MES buffer [2-(N-morpholino)ethanesulfonic acid], which provides a higher capacity at pH levels below 7 compared to Tris/HCl (Supplementary Figure 1). AUC measurements (Figure 3B) indicate an optimum pH for exonuclease activity at 7–8. Substrate degradation over time also confirmed pH 7 being the most favorable condition with a turn-over rate of 1.2 ng DNA per minute (Figure 3C), which is concordant with the duration until 50% of the substrate was degraded (Figure 3D). The amount of remaining substrate confirmed pH 7 to 8 to be the optimum condition with approximately 3% DNA left, when compared to other pH conditions with at least 5% DNA measured at the endpoint of the assay (Figure 3E).

**FIGURE 4:**
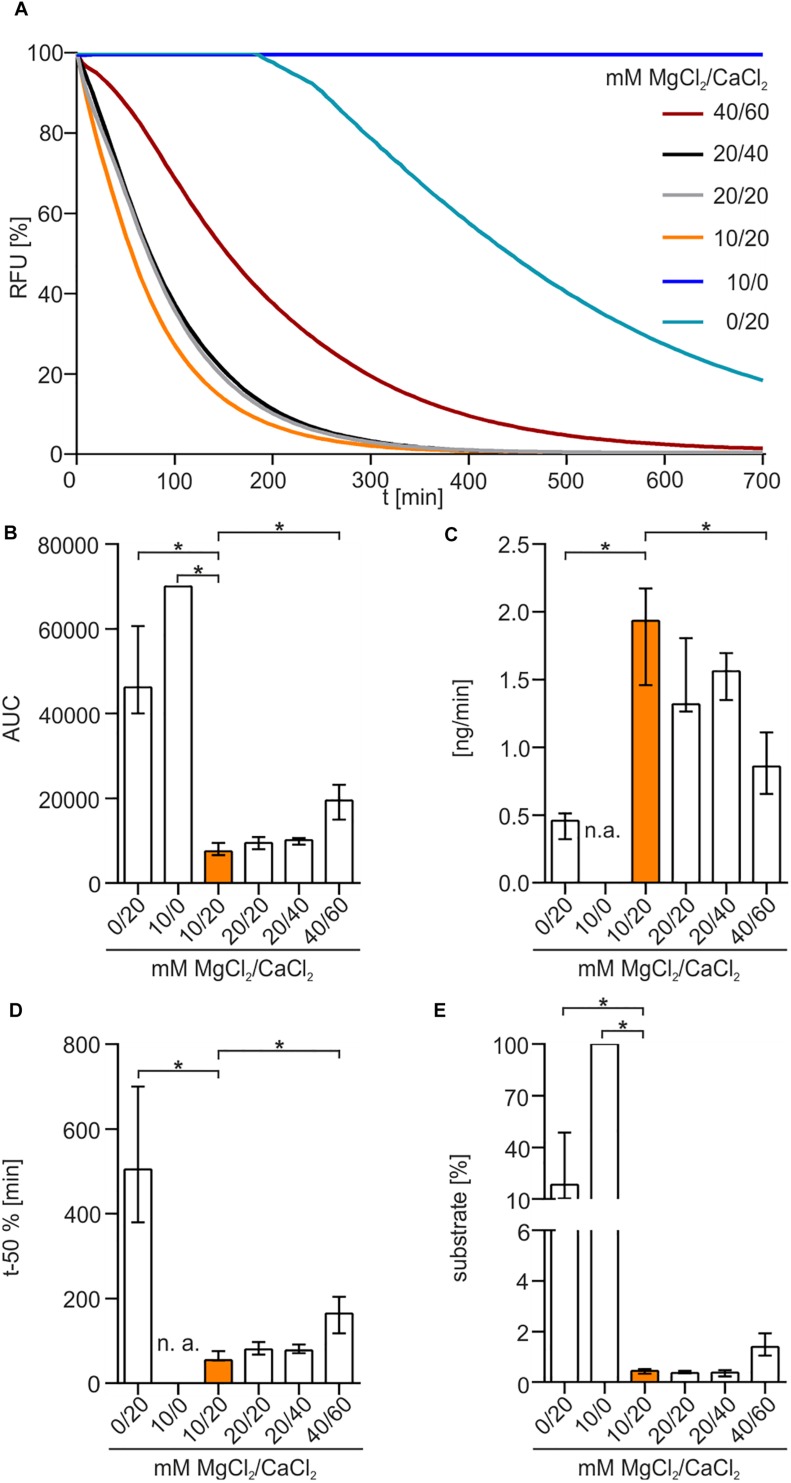
Xds activity depends on bivalent cations. **(A)** Shown are the relative fluorescence units (RFU) in percent indicating SGI bound to dsDNA. 5.1 pmol of purified Xds was incubated with 220 ng dsDNA (substrate) in buffer (50 mM Tris/HCl, pH 7; 100 mM NaCl) at 25°C with variation in concentration of MgCl_2_ and CaCl_2_. For the assay 40/60 mM MgCl_2_/CaCl_2_ (red), 20/40 mM MgCl_2_/CaCl_2_ (black), 20/20 mM MgCl_2_/CaCl_2_ (gray), 10/20 mM MgCl_2_/CaCl_2_ (orange), 10/0 mM MgCl_2_/CaCl_2_ (blue), and 0/20 mM MgCl_2_/CaCl_2_ (light blue) was used. **(B–E)** Bar charts summarize the enzyme parameters retrieved from the nuclease assays provided in panel A. Shown are the area under the curve (AUC, **B**), nanogram of substrate degraded per minute **(C)**, duration to degrade 50% of the substrate **(D)**, endpoint measurement of the remaining substrate in percent **(E)**. The data is presented as median from at least nine independent experiments. Error bars indicate the interquartile range. Significant differences to 10 mM MgCl_2_ and 20 mM CaCl_2_ are indicated by an asterisk (*P* < 0.05 Kruskal-Wallis test followed by *post hoc* Dunn’s multiple comparison). Data below limit of detections is indicated as not applicable (n. a.).

To elucidate the impact of bivalent cations on enzyme activity, different concentrations of MgCl_2_ and CaCl_2_ were used in diverse combinations (Figure 4A). No nuclease activity was detected in the absence of CaCl_2_ (Figure 4A). Presence of 20 mM CaCl_2_ without addition of MgCl_2_ showed a slow degradation of DNA after a lag-phase of approximately 3 h. In the presence of both MgCl_2_ and CaCl_2_, Xds readily degraded DNA, as highlighted by the rapid decrease in fluorescence. The optimal combination of cofactors for Xds activity was 10 mM MgCl_2_ and 20 mM CaCl_2_. This conclusion is supported by (i) a low area under curve (AUC), which reflects an efficient removal of fluorescent substrate (Figure 4B), (ii) fast degradation of DNA per minute (Figure 4C), (iii) relatively short duration until 50% of the substrate was degraded (Figure 4D) and (iv) only minor amounts of substrate remaining at the endpoint (Figure 4E).

**FIGURE 5:**
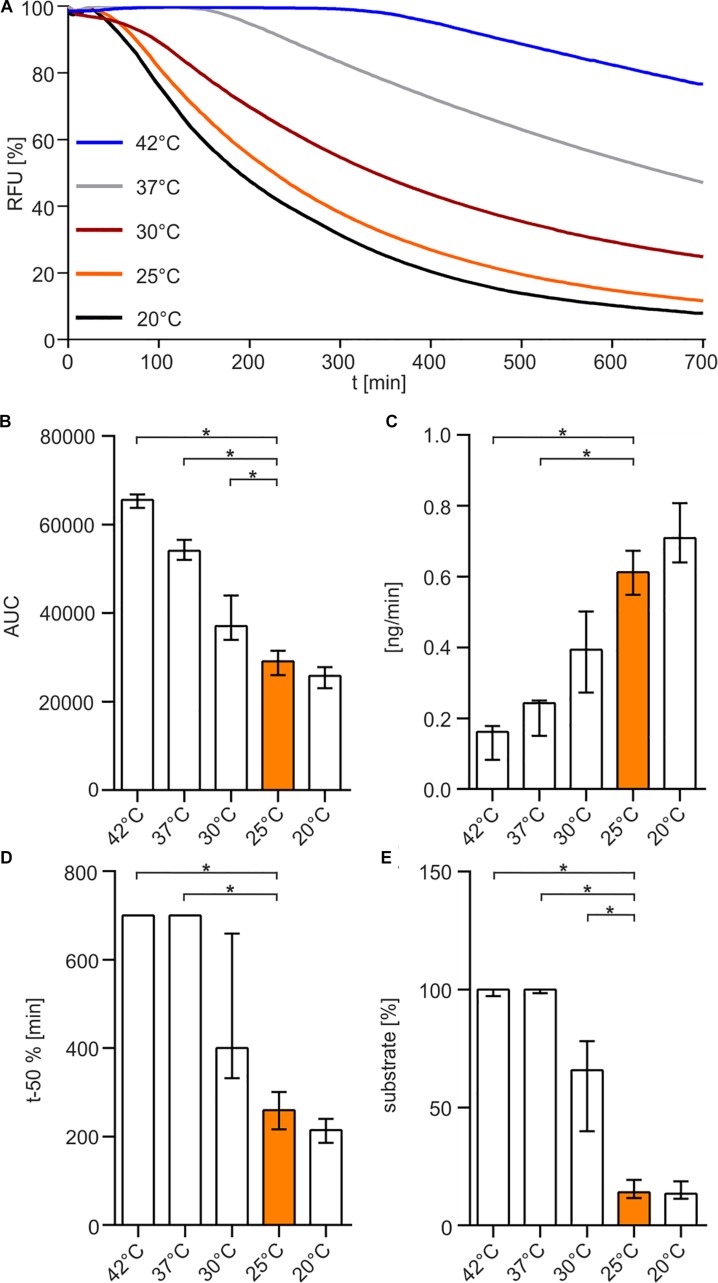
Temperature-dependency of Xds activity. **(A)** Shown are the relative fluorescence units (RFU) in percent indicating SGI bound to dsDNA. 5.1 pmol of purified Xds was pre-incubated for 30 min at different temperatures 20°C (black), 25°C (orange), 30°C (red), 37°C (gray), 42°C (blue) with 220 ng dsDNA (substrate) in Tris/HCl buffer (50 mM, pH 7). Fluorescence was measured every 5 min for 12 h. **(B–E)** Bar charts summarize the enzyme parameters retrieved from the nuclease assays provided in panel A. Shown are the area under the curve (AUC, **B**), nanogram of substrate degraded per minute **(C)**, duration to degrade 50% of the substrate **(D)**, endpoint measurement of the remaining substrate in percent **(E)**. The data is presented as median from at least 18 independent experiments. Error bars indicate the interquartile range. Significant differences to 25°C are indicated by an asterisk (*P* < 0.05 Kruskal-Wallis test followed by *post hoc* Dunn’s multiple comparison).

Finally, we investigated the temperature sensitivity of the enzyme. The enzyme was pre-incubated at 20, 25, 30, 37, or 42°C for 30 min before the substrate was added and the nuclease assay was performed at 25°C (Figure 5). The results indicate that elevated temperatures above 25°C reduce the enzymatic life time of Xds and result in a loss of enzyme activity (Figures 5A–E), highlighted by significantly increased AUC and at least 5 times more substrate remaining at the endpoint of the assay (Figures 5B,E). In addition, pre-incubation at temperatures of 37 and 42°C resulted in a significantly reduced degradation speed in the nuclease assay compared to 25°C (Figures 5C,D).

Previous studies using supernatants of *V. cholerae* deletion mutants and complementation strains indicated that Dns acts as an endonuclease, whereas Xds is only capable of degrading linearized DNA, defining it as exonuclease (Seper et al., 2011). Using the optimal buffer conditions (50 mM Tris/HCl, 100 mM NaCl, 10 mM MgCl_2_, 20 mM CaCl_2_, pH 7) in combination with the sensitive real-time nuclease activity assay we could confirm that Xds acts exclusively as an exonuclease (Supplementary Figure 2) (Seper et al., 2011). Endonuclease activity can be excluded as no degradation of circular DNA was observed (Supplementary Figure 2). Furthermore, Xds activity was assessed on linearized dsDNA substrates with differential GC content ranging from 31 to 67%. This experiment revealed a significantly faster degradation of AT-rich dsDNA compared to substrates with balanced or high GC content (Supplementary Figures 3A–E).

### Elucidation of Xds Domains Important for Exonuclease Function

Xds harbors several domains allocated to different protein families, which are connected by large, unstructured linker regions (Figure 1A). The N-terminal part of the enzyme contains a LTD (L30-G134, previously S39-G137), followed by an OB (oligonucleotide binding)-fold (N221-Q300) and the C-terminal domain (G473-I846) allocated to the EEP family (KEGG, 2017). To identify the protein parts important for nuclease activity several internal truncations of Xds were constructed. Notably, the algorithm for the identification of functional domain boundaries for Xds (Figure 1) was refined over the course of these investigations. For example, when we started to design truncations, the LTD was annotated from S39-G137 (Mitchell et al., 2015; Interpro, 2017), but recently changed to L30-G134 (Interpro, 2019; Mitchell et al., 2019). Thus, the initial truncations were designed to remove regions starting with AA S39 onward. As it will become evident below, important truncations for activity were re-constructed using the most recent algorithm for definition of the LTD domain to confirm initial results. A pre-screen allowed us to narrow down enzyme regions relevant for exonuclease activity. To this end we applied DNase test agar plates, which allow detection of extracellular DNA degradation by clearing zones surrounding the colonies, as well as simple degradation assays of a linearized PCR fragment visualized by gel electrophoresis. Based on the DNase test agar, Xds-versions truncated from AA S39 up to S184 are still able to degrade DNA, whereas protein truncations extending to the OB domain, e.g., Δ*S39-I200* showed no nuclease activity (Supplementary Figures 4A,B). The degradation assays using linearized DNA revealed that the Xds-versions lacking *S39-G137*, which comprises the predicted LTD, or *S39-Q159*, showed visible DNA degradation within 8 h compared to the nuclease mutant control (Supplementary Figure 4C). DNA degradation by both truncated proteins was slightly reduced in comparison to DNA degradation by the full-length Xds (Supplementary Figure 4C). Deletions spanning AAΔ*S39-S184* or greater showed no visible DNA degradation in this assay (Supplementary Figure 4C). Immunoblot analysis, however, revealed different expression levels of Xds full-length and the truncated versions of the protein at comparable amounts of protein (Supplementary Figures 4D,E), which might make interpretation of these results difficult.

**FIGURE 6:**
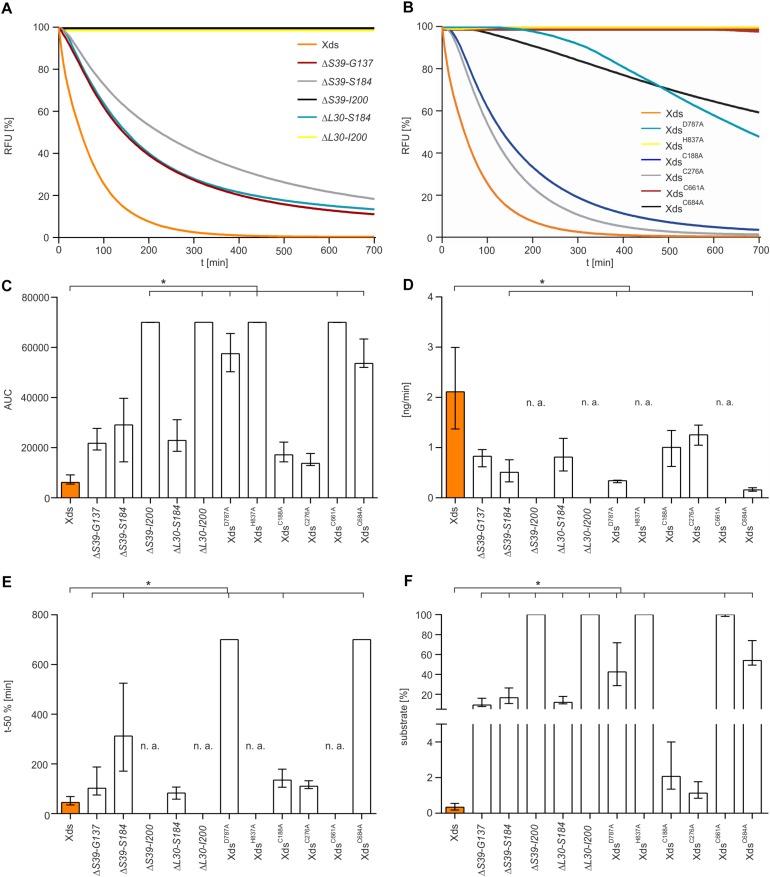
Nuclease activity of various Xds truncations and points mutants compared to full-length Xds. **(A,B)** Shown are RFU in percent indicating SGI bound to dsDNA. 5.1 pmol of purified Xds (full-length) and truncated versions of the protein were incubated with 220 ng dsDNA as a substrate. Fluorescence was measured every 5 min for 12 h in the optimal buffer conditions determined earlier (i.e., 50 mM Tris/HCl pH 7, 100 mM NaCl, 10 mM MgCl_2_, and 20 mM CaCl_2_) at 25°C. **(A)** Shown is the altered enzyme activity of the Xds truncations Δ*S39-G137* (red), Δ*S39-S184* (gray), Δ*S39-I200* (black), Δ*L30-S184* (light blue), and Δ*L30-I200* (yellow) compared to full-length Xds (orange). **(B)** Shown is the altered enzyme activity of Xds point-mutants Xds^D787A^ (light blue), Xds^H837A^ (yellow), Xds^C188A^ (blue), Xds^C276A^ (gray), Xds^C661A^ (red), and Xds^C684A^ (black) compared to full-length Xds (orange). **(C–F)** Bar charts summarize the enzyme parameters retrieved from the nuclease assays provided in panels A and B. Shown are the area under the curve (AUC, **C**), nanogram of substrate degraded per minute **(D)**, duration to degrade 50% of the substrate **(E)**, endpoint measurement of the remaining substrate in percent **(F)**. The data is presented as median from at least nine independent experiments. Error bars indicate the interquartile range. Significant differences to Xds (full-length) are indicated by an asterisk (*P* < 0.05 Kruskal-Wallis test followed by *post hoc* Dunn’s multiple comparison). Data below limit of detections is indicated as not applicable (n. a.).

Therefore, a more sophisticated assay to test nuclease activity was carried out. SGI stained dsDNA was used as substrate to measure DNA degradation by purified Xds-versions in real time in the optimal buffer condition (50 mM Tris/HCl, 100 mM NaCl, 10 mM MgCl_2_, 20 mM CaCl_2_, pH 7) (Figure 6). Most efficient degradation of DNA was observed for full-length Xds. Concordant with results obtained by assays described above, protein truncations extending to the start of the OB domain (Δ*S39-I200*) showed no activity in this assay.

As mentioned above, the LTD boundaries changed from S39-G137 to L30-G134 during the study (Mitchell et al., 2015, 2019; Interpro, 2017, 2019). To confirm that the results are also valid for the new domain annotation, several new truncations were constructed. First, we wanted to test whether the new annotated start of the LTD impacts the activity. Thus, we compared the two new truncated enzymes Δ*L30-S184* and Δ*L30-I200* to the existing Δ*S39-S184*, largest protein truncation still active, and Δ*S39-I200*, smallest truncated enzyme inactive. Nuclease activity analyzed via all three assays (DNase test agar plates, gel electrophoresis assay and the real time nuclease assay) revealed comparable activity of Δ*L30-S184* and Δ*S39-S184*, while Δ*L30-I200* shows no activity as demonstrated for Δ*S39-I200* (Supplementary Figures 5, 6). As the truncated Xds-version starting at AA L30 phenocopy the truncated protein starting at S39, the region spanning from AA L30 to S39 seems to have no significant impact on the nuclease activity.

Furthermore, we constructed several truncated enzymes to remove specific domains, i.e., Δ*L30-G134* [deletion of LTD domain according to current annotation (Interpro, 2019; Mitchell et al., 2019)], Δ*N221-Q300* (deletion of OB domain) as well as Δ*L30-G473* and Δ*S39-G473* (versions with only the EEP domain remaining). Except for the truncated protein Δ*L30-G134*, which exhibited nuclease activity similar to full length Xds, all other protein truncations (Δ*N221-Q300*, Δ*L30-G473*, and Δ*S39-G473*) showed no activity in all assays (Supplementary Figures 5, 6).

In summary, activity assays using diverse truncated versions of Xds demonstrate that the LTD domain is dispensable for nuclease activity, whereas the presence of the OB domain is required. The largest truncated protein that was still active comprises a deletion of AAL30 to S184.

### Characterization of the Active Center

**FIGURE 7:**
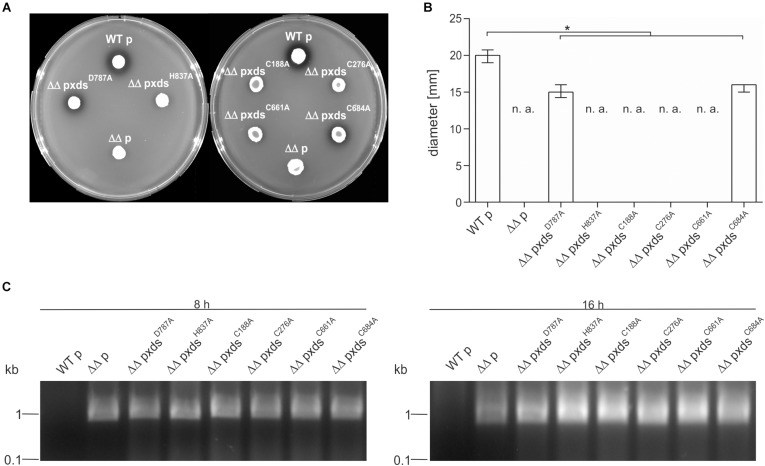
Xds point mutants exhibit different extracellular nuclease activities. **(A)** *V. cholerae* strains harboring different truncations were grown on DNase test agar and incubated with 1 N HCl after 48 h. Shown is either the WT strain C6709with empty vector (WT p), C6709Δ*xds*Δ*dns* with empty vector (ΔΔ p) or C6709Δ*xds*Δ*dns* expressing Xds point mutants as indicated. **(B)** Diameter of clearing zones are indicated for strains listed above. Shown are medians from at least 12 independent measurements. The error bars indicate the interquartile range. Significant differences between the data sets are marked by asterisk (*P* < 0.05 Kruskal-Wallis test followed by *post hoc* Dunn’s multiple comparison). Data below limit of detections is indicated as not applicable (n. a). **(C)** Supernatants derived from bacterial cultures (listed above) were assayed for their nuclease activity by adding 600 ng of linearized DNA. After incubation for 8 and 16 h the DNA degradation was visualized on agarose gels. Incubation time is indicated on top of each panel.

Additionally, we tried to pinpoint the active center of the exonuclease domain. Two AA D787 and H837, were predicted by Phyre2 analysis to form the active center when compared to other nuclease templates in the PDB. In comparison to full-length Xds, the point mutants Xds^*H*837A^ and Xds^D787A^ exhibited no or at least, severely decreased enzyme activity (Figure 6B), while immunoblot analyses revealed stable expression of the Xds point mutants (Supplementary Figure 7). These results were also confirmed by testing the mutants on DNase test agar plates, where Xds^D787A^ shows only a slight clearance zone around the expression strain, whereas for the strain expressing Xds^H837A^ no clearance could be observed (Figure 7A). Moreover, the point mutants exhibited no enzymatic activity on linearized DNA as visualized by gel electrophoresis (Figure 7C). Thus, Xds points mutants Xds^H837A^ and Xds^D787A^ are significantly impaired for nuclease activity in all assays.

Further, the AA sequence of Xds postulated four cysteine residues. C188 and C276 are in closer proximity at the N-terminal part of the protein, whereas C661 and C684 are located in the predicted exonuclease domain at the C-terminal part. Cysteine residues can from disulfide bonds which may be involved in correct folding, thereby affecting enzyme activity or their secretion [e.g., via the type 2 secretion machinery (T2SS)]. As bioinformatical analyses of Xds revealed no clear prediction on potential disulfide bond formation, all four cysteines were exchanged to alanine and their function was tested on DNase test agar plates, via gel electrophoresis (Figures 7A–C) as well as via real time nuclease assay (Figure 6B). Immunoblot analyses revealed stable expression in *V. cholerae* for all Xds point mutants (Supplementary Figure 7). No DNA degradation was detected on DNase test agar plates for strains expressing Xds^C188A^, Xds^C276A^, and Xds^C661A^, whereas a minimal zone of clearance could be detected for the strain expressing Xds^C684A^ (Figure 7A). Notably, the clearance zone of the strain expressing Xds^C684A^ was significantly smaller compared to the strain expressing Xds full-length (Figure 7B). Thus, all four Xds versions with cysteine to alanine exchanges showed less DNA degradation on DNase test agar plates. Concordant with these observations, real time degradation assays revealed impaired activity for Xds^C684A^ and no activity for Xds^C661A^ (Figures 6B–F). In contrast, Xds^C188A^ and Xds^C276A^ showed no significant difference compared to full-length Xds (Figures 6B–F). Reducing conditions using 1.4 dithiotreitol (DTT) did not affect enzyme activity in the real time nuclease assay. Thus, potential disulfide bonds formed by the cysteines seem dispensable for Xds activity (Supplementary Figure 8). Notably, detectable activity on DNase test agar plates and gel electrophoresis assay requires export of the enzyme and proper folding after secretion, which might be affected by the cysteine mutations and could explain these somewhat disparate results. Thus, we aimed to investigate the localization of Xds.

### Cellular Localization of Xds

**FIGURE 8:**
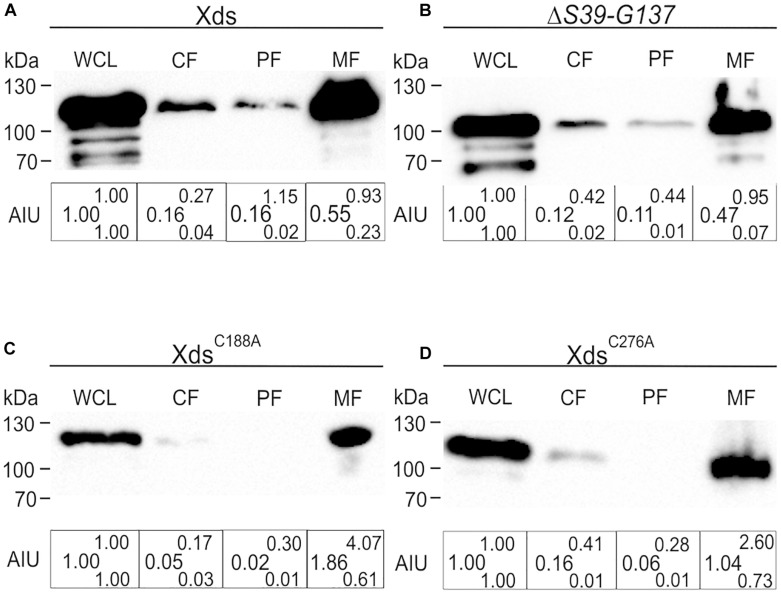
Localization of full-length Xds, the Δ*S39-G137* truncation and the cysteine point mutants Xds^C188A^ and Xds^C276A^ through fractionation. Shown are representative immunoblots detecting FLAG-tagged Xds versions in whole cell lysates (WCL), cytoplasmic fraction (CF), periplasmic fractions (PF) and membrane fraction of strain C6709Δ*xds*Δ*dns* expressing FLAG-tagged full-length Xds **(A)**, the Δ*S39-G137* truncation **(B)** or the point mutants Xds^C188A^ **(C)** and Xds^C276A^ **(D)**. Semiquantitative densitometric evaluation of detected FLAG-tagged Xds versions was performed with the Quantity One software (Bio-Rad Laboratories) and is indicated below the representative immunoblots as arbitrary intensity units (AIU) of detected FLAG-Xds normalized to WCL, which was always set to 1. At least four independent whole cell extracts of each strain were analyzed. The data is given as median with maximum (superscript) and minimum (subscript). Equal amounts of proteins for WCL, CF, PF, and MF were loaded to allow direct comparison of the fractions. SDS gels stained with Kang solution as loading control are provided in Supplementary Figure 9.

To investigate the localization of Xds in *V. cholerae* whole cell lysates (WCL), cytoplasmic fractions (CF), periplasmic fractions (PF), membrane fractions (MF) and supernatant were collected. Comparative analyses via Kang-stained gels revealed equal protein amounts for full-length Xds, the LTD deletion, as well as the two cysteine mutations analyzed (Supplementary Figures 9A–D). Samples were subjected to immunoblot analysis for the detection of FLAG-tagged proteins. Fractionation of bacteria expressing Xds full-length revealed the majority of the protein located in the WCL as well as in the MF and only a small amount in the cytoplasm and periplasm (Figure 8A). Despite several attempts using ammonium sulfate and TCA precipitated supernatant samples no detectable signal for Xds could be observed in the supernatant (data not shown). Given the presence of signal peptide for the Sec system, export via the T2SS would be the most likely option. However, a mutant of the T2SS still exhibits decent Xds activity on DNase test agar plates despite the general growth defect of T2SS mutants (Supplementary Figures 10A,B). Thus, Xds of *V. cholerae* might not be secreted into the supernatant, but rather remain associated to the bacterial surface. Hence, we focused on the residual fractions for localization analyses. *In silico* analysis of the Xds sequence revealed a N-terminal LTD (Figure 1) (KEGG, 2017). In general, LTDs of bacterial proteins are involved in membrane association, e.g., hydrolases (Krimm et al., 2002; Mans et al., 2004). Furthermore, they are frequently found in combination with oligosaccharide binding (OB) folds, especially in secreted or periplasmic bacterial proteins. This suggests a potential role for LTDs being important for tethering bacterial proteins to the membrane (Mans et al., 2004). In order to investigate this hypothesis, we constructed a truncated version lacking the LTD (S39-G137). However, subsequent fractionation revealed a similar distribution of the truncated protein as observed for full-length Xds (Figure 8B). Thus, the LTD of *V. cholerae* is not essential for membrane localization of Xds. Further, we also investigated the localization of Xds^C188A^ and Xds^C276A^ to see whether altered localization might explain the disparate results obtained with purified Xds^C188A^ and Xds^C276A^ in the real time nuclease activity assay and DNase test agar plates (Figures 6, 7). Based on the fractionation results, Xds^C188A^ and Xds^C276A^ are apparently not impaired in their localization as they exhibit a similar localization as full-length Xds (Figures 7A,B, 8C,D). Thus, Xds^C188A^ and Xds^C276A^ might show improper folding or reduced stability in *V. cholerae* explaining the negative result on the DNase test agar and by gel electrophoresis assay.

## Discussion

The extracellular nuclease Xds has been recently reported to play key roles in colonization fitness of *V. cholerae* and biofilm formation (Seper et al., 2011, 2013; Gumpenberger et al., 2016). These findings suggest that Xds could be a potential target for therapy, as it is only present in a small set of bacteria and no human homologs exist. As a first step, this study characterizes Xds of *V. cholerae* with regard to its enzymatic properties and active center. Real time nuclease assay, performed with SGI stained dsDNA, allowed the measurement of DNA degradation by the enzyme over a time period of 12 h. Despite the fact that *V. cholerae* can cope with high salinity, the optimal NaCl concentration for Xds was found to be 100 mM or lower. This result is consistent with the previously reported optimal 175 mM NaCl concentration for Dns endonuclease; a value closer to brackish water than seawater (Altermark et al., 2007). Additionally, studies show that biofilm formation is the highest in medium with a salt concentration of 100 mM NaCl, and which becomes less with decrease or increase of osmolarity (Shikuma et al., 2009). Increasing salt concentration leads to a decrease of Dns activity (Altermark et al., 2007), which we could also confirm for Xds.

The optimum pH for the exonuclease was shown to be at pH 7 to 8, which is consistent with the optimum pH for Dns activity (Altermark et al., 2007). Interestingly, fluctuation in pH does not significantly affect protein activity when compared to variations in salt concentration. Thus, Xds activity is resistant to a wide range of pH levels. Considering the importance of Xds during biofilm formation in the aquatic environment (pH 9) and Xds contribution to bacterial escape from NETs in the upper small intestine (pH 6–7.4), the adaptation of the enzyme to a wide range of pH present in both environments reflects versatile physiological roles (Fallingborg, 1999; Hostacka et al., 2010). Furthermore, presence of bivalent cations, such as MgCl_2_ and CaCl_2_, is crucial for Xds activity. Maximum activity was only obtained when both cations were present, indicating that they act synergistically for Xds activity and/or stability. Importance of Mg^2+^ for full activation of exonuclease function has been previously shown for the nuclease domain of CNOT6L, which was also used for our sequence homology model, putatively identifying the active center of the enzyme (Wang et al., 2010). Additionally, previous studies on extracellular nucleases of *Pseudomonas* indicated that exonuclease activity is dependent on both Ca^2+^ and Mg^2+^ ions (Gray, 1975; Gray et al., 1975), which was herein confirmed for Xds.

Using the optimized buffer conditions, several additional enzyme characteristics were elucidated. Purified Xds seems to be heat-sensitive as incubation temperatures above 30°C shortened the life time of the enzyme. Variation of the GC content of the linear dsDNA substrate revealed a higher Xds activity for AT-rich fragments. We speculate that the weaker stability of the A/T hydrogen bonding allows an easier strand separation and results in faster hydrolysis of the DNA to nucleotides. Consistent with a previous report (Seper et al., 2011), the nuclease assay used herein confirmed that Xds has exonuclease activity only capable of degrading linear DNA but not circular DNA. In contrast ExeM of *Shewanella oneidensis*, a homolog of Xds with 41% identity, was recently characterized to have sugar-unspecific endonuclease activity (Binnenkade et al., 2018). With 94.3 kDa Xds represents a relatively high molecular weight multi-domain enzyme. To characterize the importance of different parts of the protein for Xds activity, several in-frame deletions were constructed. Nuclease activity assays using diverse truncated protein versions demonstrate that the largest truncation that still allowed enzymatic activity spans from AA L30 to S184, while deletion of an additional 15 AA (including I200 and beyond) results in an inactive nuclease. Thus, the presence of the OB and EEP domain is required for activity, whereas the N-terminal LTD seems to be dispensable. LTDs are often associated with OB domains, important for binding of nucleotide sequences, which is consistent with the Xds model (Murzin, 1993; Mans et al., 2004). We speculated that the LTD domain could be important for tethering Xds to the membrane as suggested for other bacterial proteins (Krimm et al., 2002; Mans et al., 2004). However, the LTD-truncated Xds showed no significant difference in its localization compared to full-length Xds. Thus, the LTD of Xds seems to be dispensable for its membrane association. In addition, at least 20 AA on the N-terminal side of the OB domain are also required for activity. Large AA stretches linking the LTD with the OB domain and the nuclease domain are predicted to be unstructured, which might indicate that this region is reorganized after binding DNA as substrate.

Moreover, we constructed point mutations in two residues predicted to lie within the enzymatic active center by *in silico* analyses, changing D787 and H837 to alanine. Real time activity assays elucidated that Xds^D787A^ is heavily impaired for DNA degradation and Xds^H837A^ shows no nuclease activity. The H837 might have a crucial role to act as the general base in activation of the water molecule for the nucleophilic in-line attack on the phosphorous atom during the nuclease reaction as suggested by other studies (Bueren-Calabuig et al., 2011; Oliva et al., 2017). Mechanisms of substrate binding as well as the catalytic process focusing on the predicted active site residues have to be further investigated but we herein confirm that both AA play an important role in the DNase activity of the protein. Furthermore, we tried to elucidate the role of the four cysteines that are present in the Xds sequence. Supernatants of *V. cholerae* strains expressing point mutants of the four cysteines exhibited no visible exonuclease activity on linear dsDNA. Concordantly, strains expressing Xds^C188A^, Xds^C276A^, Xds^C661A^, and Xds^C684A^ showed no or at least significantly reduced clearance zones on DNase test agar plates compared to Xds full-length. The real time nuclease assay with purified protein confirmed minor degradation of dsDNA for Xds^C661A^ and Xds^C684A^, whereas Xds^C188A^ and Xds^C276A^ showed similar activity to Xds full-length. Bioinformatic analyses revealed no clear prediction on potential disulfide bonds by the cysteines. Notably, addition of the reducing agent DTT to the real time nuclease activity assay did not affect nuclease activity of Xds, excluding a disulfide bond formation for its enzymatic function. However, disulfide bond formation could be important for secretion as well as correct folding along or after transport through the outer membrane. Fractionation of both mutant strains showed FLAG tagged protein in WCL, minor signal in CF and almost no signal in PF. In fact there was almost no difference comparing protein amount in WCL and MF, which indicates proteins are not subjected to massive degradation due to incorrect folding. We therefore hypothesize that Xds^C188A^ and Xds^C276A^ are impaired for proper folding along or after the secretion process. This would explain the observed discrepancy of these point mutants showing decent activity in nuclease assays using purified protein, but no activity of the respective strains expressing the point mutants on the DNase test agar plate assay.

The present work characterized the *V. cholerae* extracellular nuclease Xds with regard to the optimum reaction conditions including pH, salt concentration, bivalent cations, and temperature as well as identification of a domain within the protein important for its enzymatic activity. This is a first step toward a better understanding of the enzymatic properties of Xds affecting *V. cholerae’s* survival fitness in the aquatic environment as well as colonization fitness in the human host.

## Data Availability

All datasets generated for this study are included in the manuscript and/or the Supplementary Files.

## Author Contributions

KP, MO, JR, and SS designed the study. KP, FM, DV, and MO performed the experiments and/or the analysis. KP, FM, DV, MO, JR, and SS contributed to the discussion and data evaluation. KP, MO, JR, and SS wrote the manuscript.

## Conflict of Interest Statement

The authors declare that the research was conducted in the absence of any commercial or financial relationships that could be construed as a potential conflict of interest.
