# Supplementary material for: Characterization of Vibrio cholerae’s Extracellular Nuclease Xds
Source: Front Microbiol. 2019 Sep 10;10:2057. doi: 10.3389/fmicb.2019.02057 (PMC6746945; doi:10.3389/fmicb.2019.02057)
Supplement: Supplementary file 1 [file Data_Sheet_1.PDF]

Supplementary Material

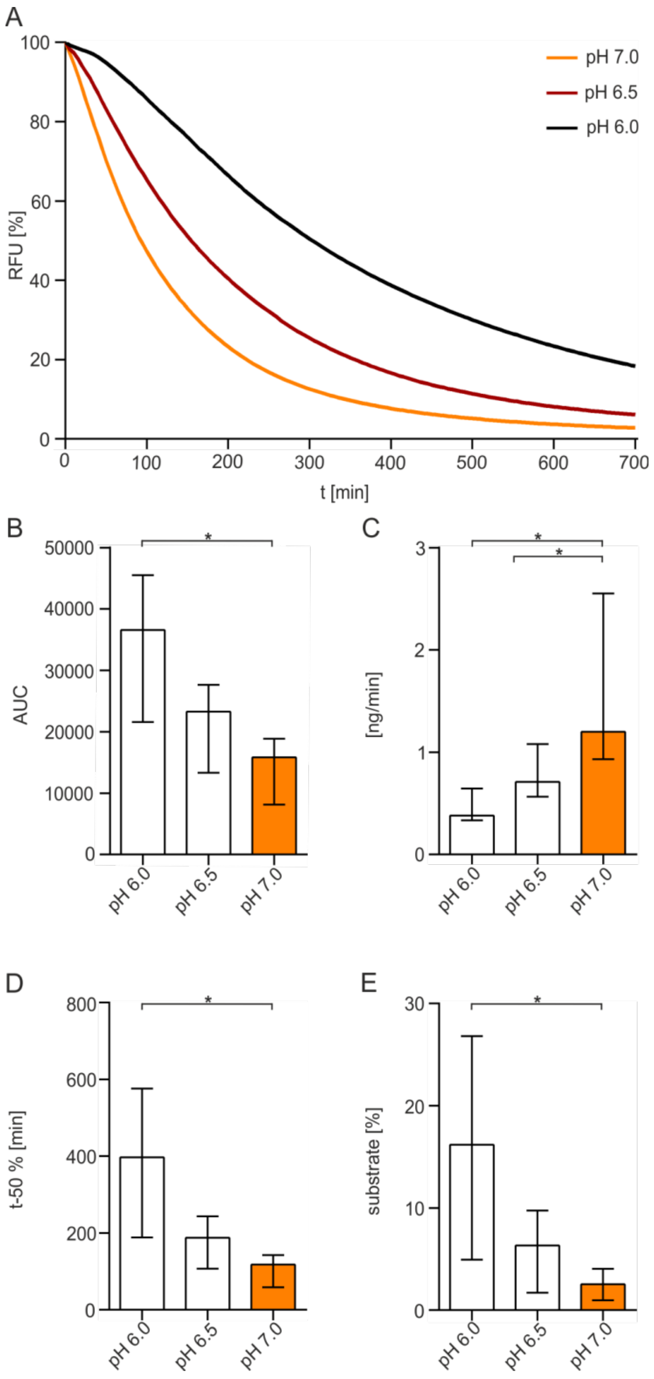

Supplemental Figure 1. pH-dependency of Xds activity in MES buffer. (**A**) Shown are the RFU in percent indicating SGI bound to dsDNA. 5.1 pmol of purified Xds was incubated with 220 ng

dsDNA (substrate) in 2-(N-morpholino)ethanesulfonic acid buffer (50 mM) at 25°C with variation in pH, pH 7.0 (orange), pH 6.5 (red) and pH 6.0 (black). Fluorescence was measured every five minutes for 12 h. (**B-E**) Bar charts summarize the enzyme parameters retrieved from the nuclease assays provided in panel A. Shown are the area under the curve (AUC, **B**), nanogram of substrate degraded per minute (**C**), duration to degrade 50% of the substrate (**D**), endpoint measurement of the remaining substrate in percent (**E**). The data is presented as median from at least 12 independent experiments. Error bars indicate the interquartile range. Significant differences to pH 7.0 are indicated by an asterisk ($P < 0.05$ Kruskal-Wallis test followed by post hoc Dunn's multiple comparison).

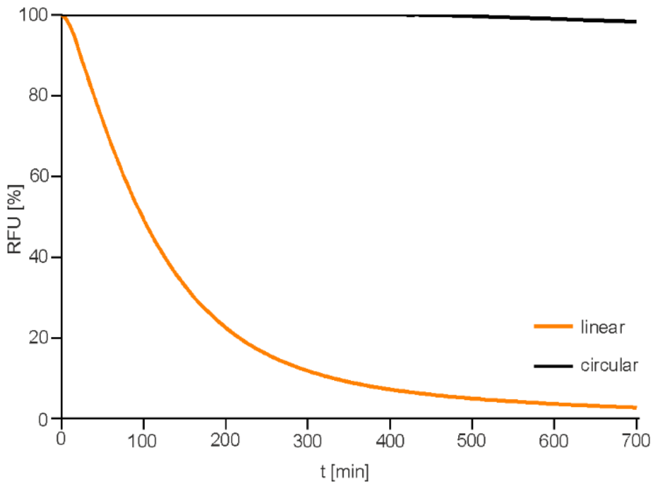

Supplemental Figure 2. Xds exhibits solely exonuclease activity. Shown are the relative fluorescence units (RFU) in percent indicating SGI bound to dsDNA. 5.1 pmol of purified Xds protein was incubated with 220 ng dsDNA as a substrate. dsDNA was used as linear (orange) or circular (black) from. Fluorescence was measured every five minutes for 12 h in the optimal buffer conditions determined earlier (i.e. 50 mM Tris/HCl pH 7, 100 mM NaCl, 10 mM $MgCl_2$ and 20 mM $CaCl_2$) at 25°C. The data is presented as median from at least 9 independent experiments.

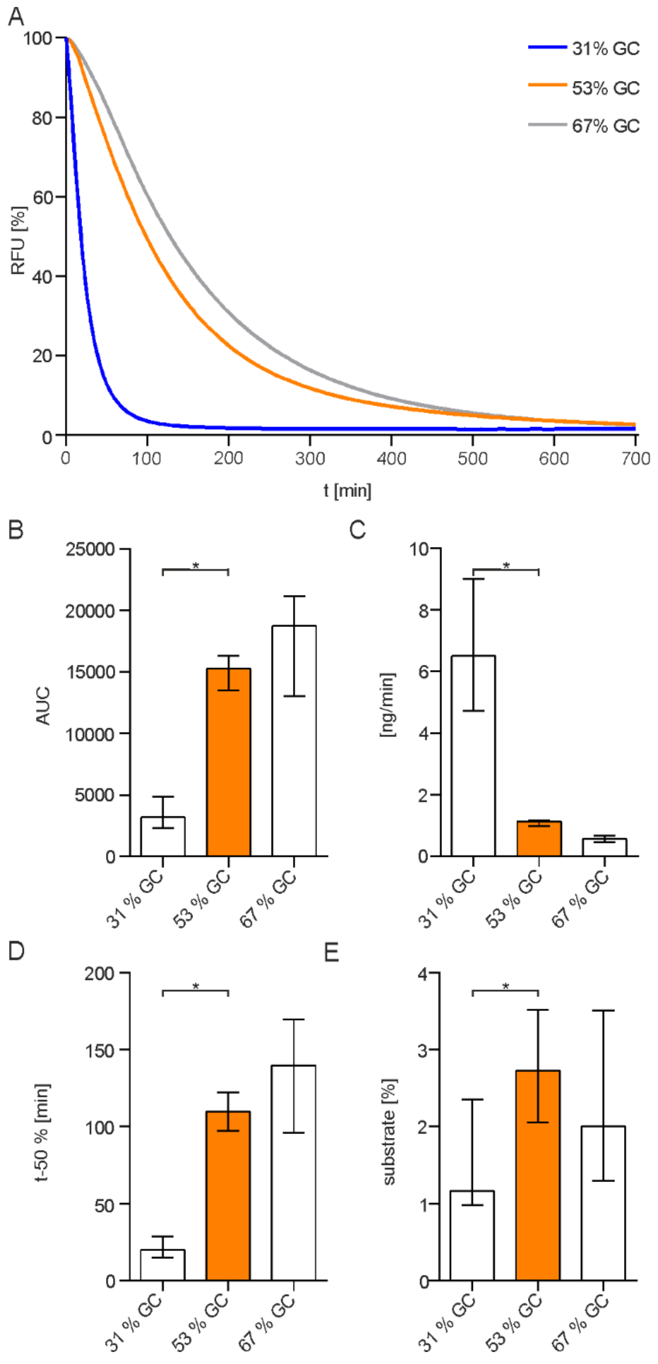

Supplemental Figure 3. Impact of GC content on Xds. Shown are RFU in percent indicating SGI bound to dsDNA. 5.1 pmol of purified Xds protein was incubated with 220 ng dsDNA as a substrate. Fluorescence was measured every five minutes for 12 h in the optimal buffer conditions determined earlier (i.e. 50 mM Tris/HCl pH 7, 100 mM NaCl, 10 mM $MgCl_2$ and 20 mM $CaCl_2$) at 25°C. (**A**)

Shown are the enzyme activities of the Xds on dsDNA with varying GC content, 31% (blue), 53% (orange) and 67% (grey). (**B-E**) Bar charts summarize the enzyme parameters retrieved from the nuclease assays provided in panel A. Shown are the area under the curve (AUC, **B**), nanogram of substrate degraded per minute (**C**), duration to degrade 50% of the substrate (**D**), endpoint measurement of the remaining substrate in percent (**E**). The data is presented as median from at least 12 independent experiments. Error bars indicate the interquartile range. Significant differences to 53% GC (standard dsDNA) are indicated by an asterisk ($P < 0.05$ Kruskal-Wallis test followed by post hoc Dunn's multiple comparison).

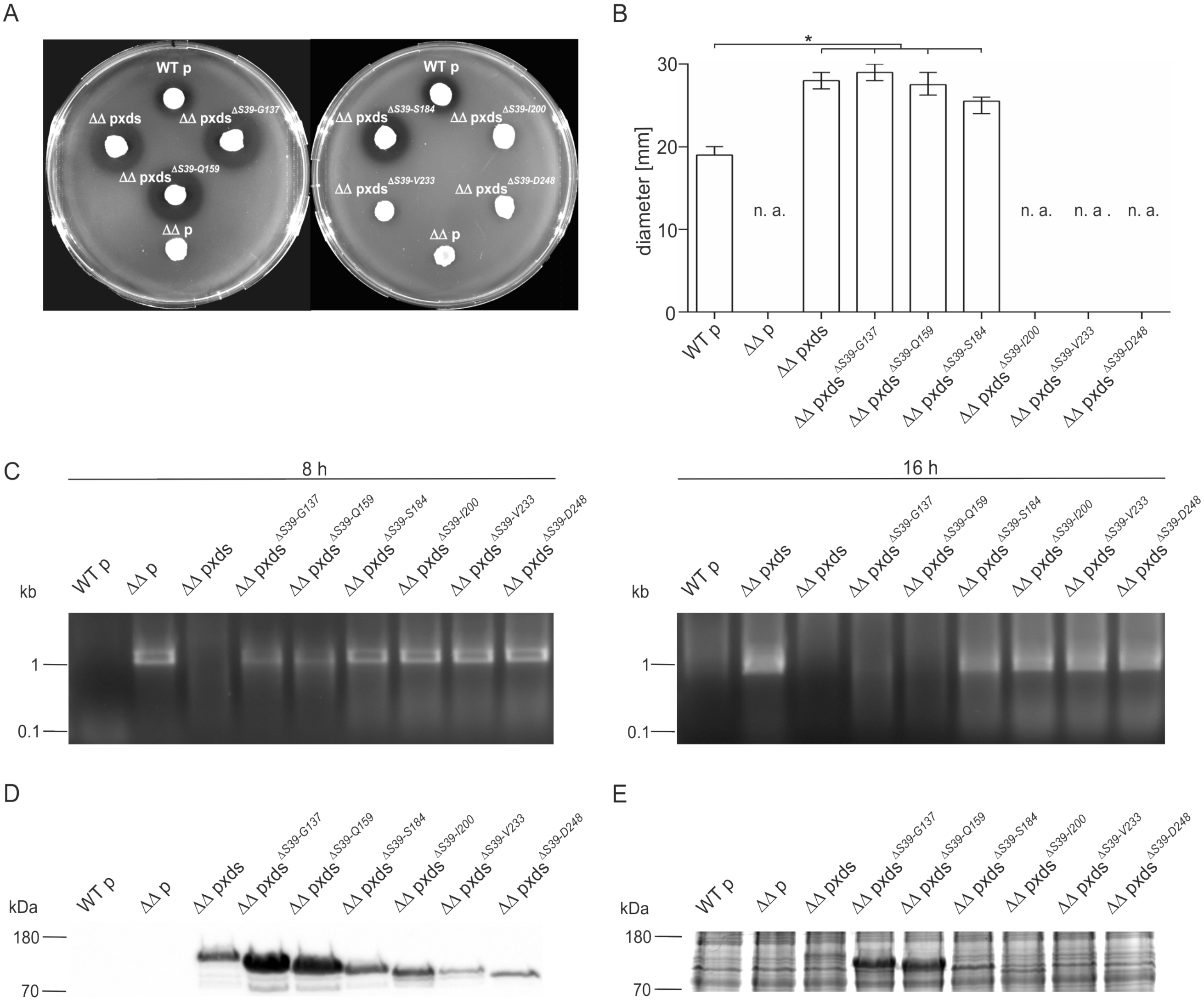

Supplemental Figure 4. AA S39 to S184 are dispensable for extracellular nuclease activity of Xds in *V. cholerae*. Strains used in these assays are WT strain C6709 with empty vector (WT p) as well as C6709Δ*xds*Δ*dns* harboring the empty vector (ΔΔ p), expression plasmids for full-length Xds (ΔΔ pxds) or truncated Xds versions (ΔΔ pxds$^{\Delta S39\text{-}Y}$, whereby Y stands for the last AA missing). (**A**) *V. cholerae* strains harboring different Xds-truncations were grown on DNase test agar and incubated with 1 N HCl after 48 h.(**B**) Diameter of clearing zones of the *V. cholerae* strains harboring different Xds-truncations as indicated on the x-axis. Shown are medians from at least 12 independent measurements. The error bars indicate the interquartile range. Significant differences between the

data sets are marked by asterisk ($P < 0.05$ Kruskal-Wallis test followed by *post hoc* Dunn's multiple comparison). Data below limit of detections is indicated as not applicable (n.a.). (**C**) Supernatants derived from bacterial cultures were assayed for their nuclease activity by adding 600 ng of linearized DNA. DNA degradation was visualized on agarose gels after incubation for 8 and 16 h, respectively. Incubation time is indicated on top if each panel. (**D**) Immunoblot analysis of whole cell lysates obtained from different strains harboring FLAG-tag are indicated to visualize the expression levels of Xds full-length and the truncated version of the protein. (**E**) Comparative analysis of protein amounts visualized on a SDS gel stained with Kang solution as loading control for the immunoblot analysis.

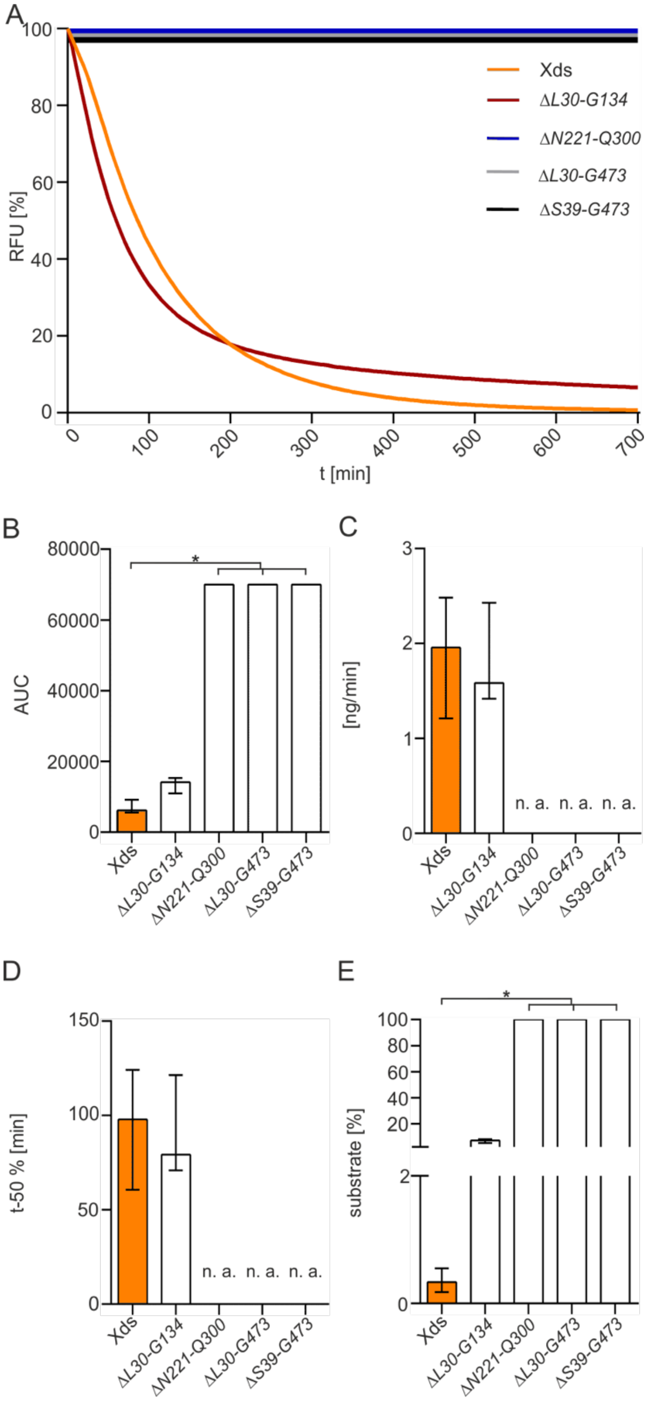

Supplemental Figure 5. Impact of protein domains of Xds on nuclease activity. (**A, B**) Shown are RFU in percent indicating SGI bound to dsDNA. 5.1 pmol of purified Xds (full-length) and truncated versions of the protein were incubated with 220 ng dsDNA as a substrate. Fluorescence was measured every five minutes for 12 h in the optimal buffer conditions determined earlier (i.e. 50 mM

Tris/HCl pH 7, 100 mM NaCl, 10 mM $MgCl_2$ and 20 mM $CaCl_2$) at 25°C. (**A**) Shown is the altered enzyme activity of the Xds truncations Δ*L30-G134* (red), Δ*N221-Q300* (blue), Δ*L30-G473* (grey) and Δ*S39-G473* (black) and compared to full-length Xds (orange). (**B-E**) Bar charts summarize the enzyme parameters retrieved from the nuclease assays provided in panel A. Shown are the area under the curve (AUC, **B**), nanogram of substrate degraded per minute (**C**), duration to degrade 50% of the substrate (**D**), endpoint measurement of the remaining substrate in percent (**E**). The data is presented as median from at least 9 independent experiments. Error bars indicate the interquartile range. Significant differences to Xds (full-length) are indicated by an asterisk ($P < 0.05$ Kruskal-Wallis test followed by post hoc Dunn's multiple comparison). Data below limit of detections is indicated as not applicable (n. a.).

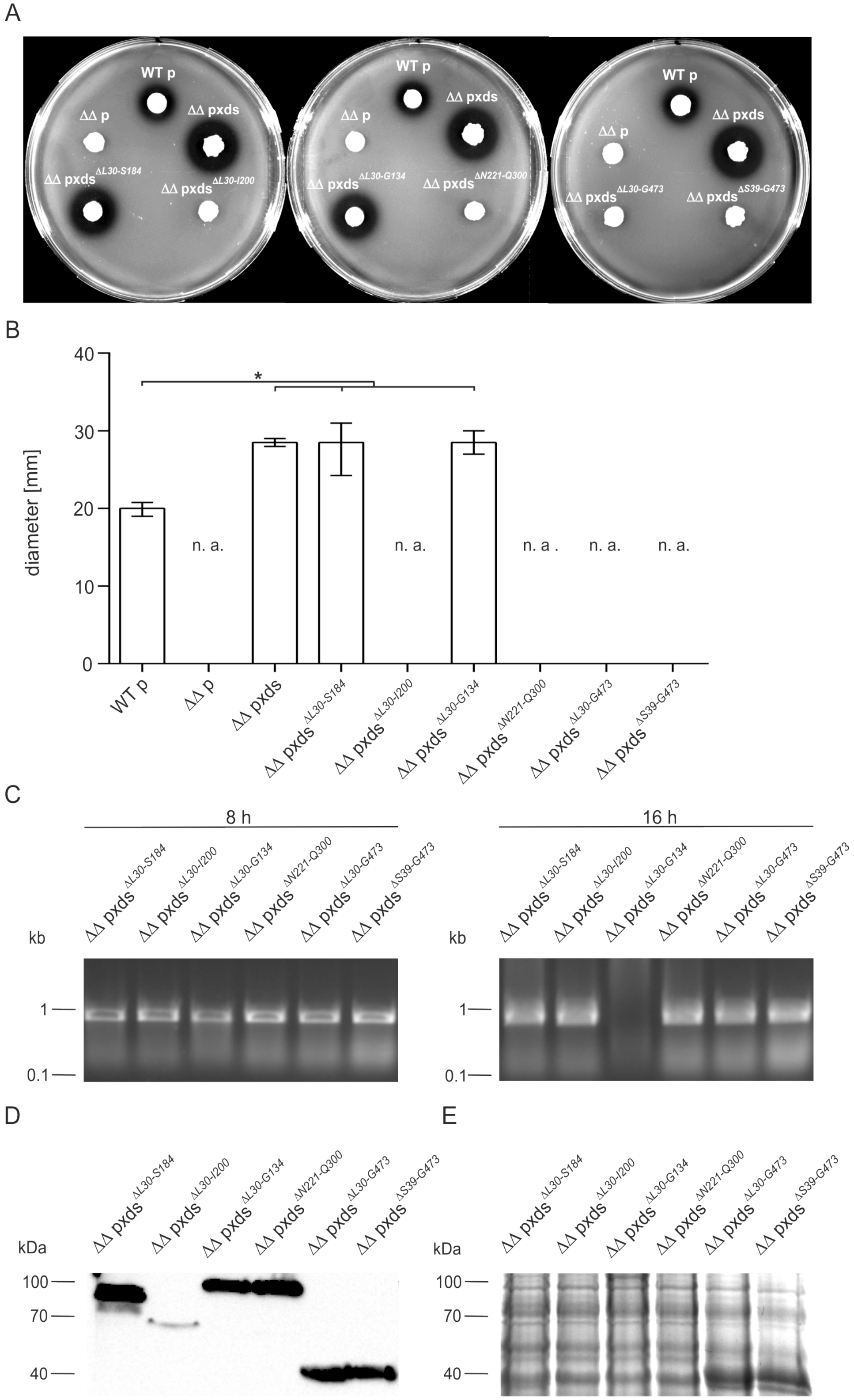
A
WT p
ΔΔ p
ΔΔ pxds
ΔΔ pxds ΔL30-S184
ΔΔ pxds ΔL30-I200
ΔΔ pxds ΔL30-G134
ΔΔ pxds ΔN221-Q300
ΔΔ pxds ΔL30-G473
ΔΔ pxds ΔS39-G473
B
diameter [mm]
0
10
20
30
40
*
n. a.
WT p
ΔΔ p
ΔΔ pxds
ΔΔ pxds ΔL30-S184
ΔΔ pxds ΔL30-I200
ΔΔ pxds ΔL30-G134
ΔΔ pxds ΔN221-Q300
ΔΔ pxds ΔL30-G473
ΔΔ pxds ΔS39-G473
C
8 h
16 h
kb
1
0.1
D
E
kDa
100
70
40

Supplemental Figure 6. Amino acid (AA) region from L30 to S39 and LTD domain, but not the OB domain, are dispensable for extracellular nuclease activity of Xds in *V. cholerae*. Strains used in these assays are WT strain C6709 with empty vector (WT p) as well as C6709Δ*xds*Δ*dns* harboring the empty vector (ΔΔ p), expression plasmids for full-length Xds (ΔΔ pxds) or truncated Xds-versions (ΔΔ $pxds^{\Delta X\text{-}Y}$, whereby X stands for the first and Y for the last AA missing). (**A**) *V. cholerae* strains harboring different Xds-truncations were grown on DNase test agar and incubated with 1 N HCl after 48 h. (**B**) Diameter of clearing zones of the *V. cholerae* strains harboring different Xds-truncations on the DNase test agar plates are indicated. Shown are medians from at least 12 independent measurements. The error bars indicate the interquartile range. Significant differences between the data sets are marked by asterisk ($P < 0.05$ Kruskal-Wallis test followed by *post hoc* Dunn's multiple comparison). Data below limit of detections is indicated as not applicable (n.a.). (**C**) Supernatants derived from bacterial cultures (listed above) were assayed for their nuclease activity by adding 600 ng of linearized DNA. DNA degradation was visualized on agarose gels after incubation for 8 and 16 h, respectively. Incubation time is indicated on top if each panel. (**D**) Immunoblot analysis of whole cell lysates obtained from different strains harboring FLAG-tag are indicated to visualize the expression levels of Xds full-length and the truncated version of the protein. (**E**) Comparative analysis of protein amounts visualized on a SDS gel stained with Kang solution as loading control for the immunoblot analysis.

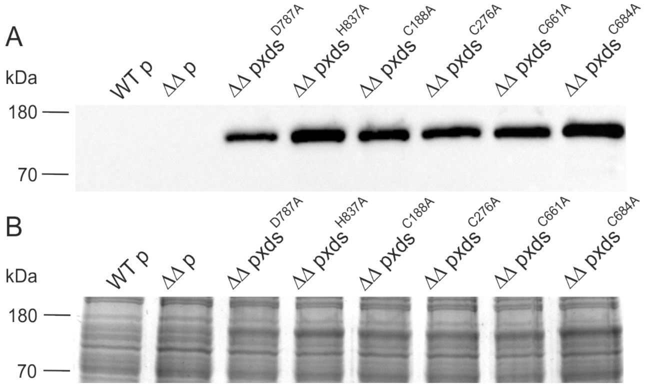

Supplemental Figure 7. Expression levels of WCL representing Xds point mutants. (**A**) Immunoblot analysis of WCL obtained from different strains harboring empty vector [WT strain C6709 with empty vector (WT p) and C6709Δ*xds*Δ*dns* harboring the empty vector (ΔΔ p)] or C6709Δ*xds*Δ*dns* harboring expression plasmids for FLAG-tagged Xds point mutants (ΔΔ pxds^{x}, whereby ^{X} stands for the point mutation) are indicated to visualize their expression levels. (**B**) Comparative analysis of protein amounts visualized on a SDS gel stained with Kang solution as loading control for the immunoblot analysis.

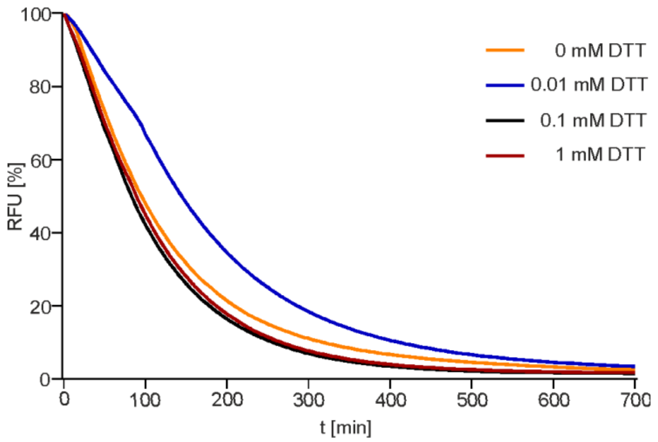

Supplemental Figure 8. DTT has no impact on Xds activity. Shown are RFU in percent indicating SGI bound to dsDNA. 5.1 pmol of purified Xds protein was incubated with 220 ng dsDNA as a substrate. Fluorescence was measured every five minutes for 12 h in the optimal buffer conditions determined earlier (i.e. 50 mM Tris/HCl pH 7, 100 mM NaCl, 10 mM $MgCl_2$ and 20 mM $CaCl_2$) at 25°C with a variation in 1.4-Dithiothreitol concentration, 0 mM DTT (orange), 0.01 mM DTT (blue), 0.1 mM DTT (black) and 1 mM DTT (red). The data is presented as median from at least 12 independent experiments.

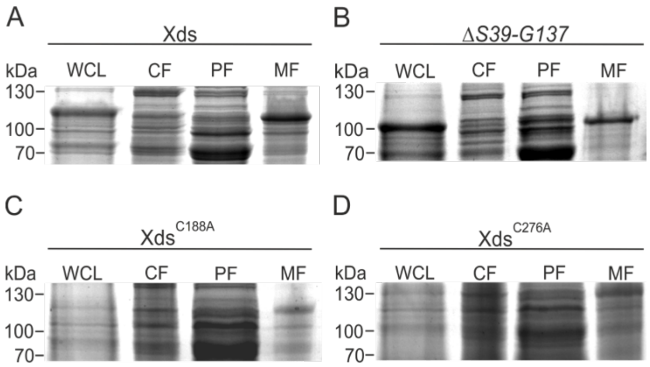

Supplemental Figure 9. Comparative analysis of protein amounts visualized on a SDS gel stained with Kang solution as loading control for the immunoblot analysis. Shown are the protein amounts of fractions, whole cell lysates (WCL), cytoplasmic fraction (CF), periplasmic fractions (PF) and membrane fraction (MF) of strain C6709Δ*xds*Δ*dns* expressing FLAG-tagged full-length Xds (**A**), the Δ*S39-G137* truncation (**B**) or the point mutants Xds^{C188A} (**C**) and Xds^{C276A} (**D**) serving as loading control for immunoblot displayed in Figure 8.

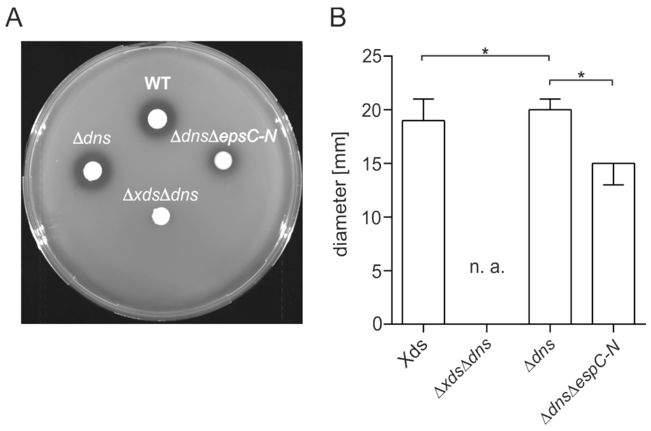

Supplemental Figure 10. Δ*epsC-N* mutants (deficient for T2SS) still exhibit extracellular Xds activity. (**A**) *V. cholerae* strains C6709 (WT) and indicated deletion mutants were grown on DNase test agar and incubated with 1 N HCl after 48 h. (**B**) Diameter of clearing zones of the *V. cholerae* strains on the DNase test agar plates are indicated. Shown are medians from at least 11 independent measurements. The error bars indicate the interquartile range. Significant differences between the data sets are marked by asterisk ($P < 0.05$ Kruskal-Wallis test followed by *post hoc* Dunn's multiple comparison). Data below limit of detections is indicated as not applicable (n. a.).
